# Spatiotemporal single‐cell transcriptomic profiling reveals inflammatory cell states in a mouse model of diffuse alveolar damage

**DOI:** 10.1002/EXP.20220171

**Published:** 2023-04-10

**Authors:** Duo Su, Zhouguang Jiao, Sha Li, Liya Yue, Cuidan Li, Mengyun Deng, Lingfei Hu, Lupeng Dai, Bo Gao, Jinglin Wang, Hanchen Zhang, Haihua Xiao, Fei Chen, Huiying Yang, Dongsheng Zhou

**Affiliations:** ^1^ State Key Laboratory of Pathogen and Biosecurity Beijing Institute of Microbiology and Epidemiology Beijing China; ^2^ Reproductive Genetics Center Bethune International Peace Hospital Shijiazhuang China; ^3^ State Key Laboratory of Biochemical Engineering, Institute of Process Engineering Chinese Academy of Sciences Beijing China; ^4^ School of Basic Medical Sciences Anhui Medical University Hefei China; ^5^ Laboratory of Genome Sciences & Information, Beijing Institute of Genomics Chinese Academy of Sciences and China National Center for Bioinformation Beijing China; ^6^ Beijing National Laboratory for Molecular Science, State Key Laboratory of Polymer Physical and Chemistry Institute of Chemistry, Chinese Academy of Science Beijing China

**Keywords:** diffuse alveolar damage, fibroblast, neutrophil, single‐cell RNA sequencing, spatial transcriptomics

## Abstract

Diffuse alveolar damage (DAD) triggers neutrophilic inflammation in damaged tissues of the lung, but little is known about the distinct roles of tissue structural cells in modulating the recruitment of neutrophils to damaged areas. Here, by combining single‐cell and spatial transcriptomics, and using quantitative assays, we systematically analyze inflammatory cell states in a mouse model of DAD‐induced neutrophilic inflammation after aerosolized intratracheal inoculation with ricin toxin. We show that homeostatic resident fibroblasts switch to a hyper‐inflammatory state, and the subsequent occurrence of a CXCL1‐CXCR2 chemokine axis between activated fibroblasts (AFib) as the signal sender and neutrophils as the signal receiver triggers further neutrophil recruitment. We also identify an anatomically localized inflamed niche (characterized by a close‐knit spatial intercellular contact between recruited neutrophils and AFib) in peribronchial regions that facilitate the pulmonary inflammation outbreak. Our findings identify an intricate interplay between hyper‐inflammatory fibroblasts and neutrophils and provide an overarching profile of dynamically changing inflammatory microenvironments during DAD progression.

## Graphical abstract

Diffuse alveolar damage (DAD) triggers intense neutrophilic airway inflammation. This work demonstrates that proinflammatory activated fibroblasts (AFib) are induced in a mouse model of DAD. Neutrophil infiltration into the airway crucially depends on their cross‐talk with AFib via the soluble CXCL1‐CXCR2 chemokine axis.

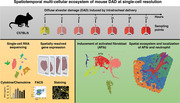

## 1 INTRODUCTION

Diffuse alveolar damage (DAD) is an acute lung insult caused by smoke or toxic inhalant that results in pneumonia, a cytokine storm, and massive neutrophil infiltration.^[^ ^1^ ^]^ To date, therapeutic strategies for this intractable disease are lacking due to an insufficient understanding of its pathogenesis. Therefore, exploration of its key cellular subpopulations and their activated states in the lung tissue microenvironment is important for discovering new treatment targets of DAD.

Basic research in animal models of DAD has provided insight into immunopathological mechanisms,^[^ ^2^ ^]^ revealing the role of neutrophilic infiltration in driving the inflammatory response and subsequent inflammatory burst that eventually compromises lung function.^[^ ^3^ ^]^ Our previous study revealed some underlying pathogenic molecular mechanisms (such as leukocyte migration and activated immunoregulation‐associated transcriptional factors) of ricin‐induced DAD through “bulk” RNA sequencing (RNA‐seq) analyses.^[^ ^4^ ^]^ Despite these observations, the critical question of what drives the early burst of pulmonary inflammation remains unanswered. The heterogeneity of the pulmonary cell atlas and the interaction of different cell types in the microenvironment are still poorly understood due to a lack of single‐cell resolution technology.

Recent advances in single‐cell RNA‐seq (scRNA‐seq) and spatial transcriptomics (STomics) provide powerful new tools for massive parallel delineation of cell states, interactions, and spatial distribution in vivo. Compared to conventional RNA‐seq, scRNA‐seq allows tracking changes in individual cell populations at the single‐cell level in an unbiased manner.^[^ ^5^ ^]^ Moreover, integrating scRNA‐seq with STomics enables the profiling of spatially resolved local cellular heterogeneity, revealing the ecosystem of cellular components and the complexities of molecular interactions in a progressive disease. Increasingly, proof‐of‐concept studies have demonstrated the feasibility and advantage of these approaches in elucidating the molecular pathogenesis of multiple disease‐associated cells in clinical organ samples.^[^ ^6^ ^]^

In this study, we integrate scRNA‐seq with STomics to temporally and spatially characterize the pulmonary cellular microecosystems in a mouse model of DAD after aerosolized intratracheal inoculation of ricin toxin. Our research reveals the dynamic changes of cellular and inflammatory mediators during DAD progression. Fibroblasts underwent remarkable remodeling, shifting toward pro‐inflammatory phenotypes, and excess activated fibroblasts (AFib) were spatially enriched with neutrophils, which facilitated the relocation of neutrophils into the parenchyma, thus increasing inflammation. This work provides insight into the immune‐boosting signaling pattern between resident lung fibroblasts and recruited neutrophils during DAD progression, raising the possibility of targeting intercellular crosstalk to reduce pulmonary inflammatory progression.

## 2 METHODS AND RESULTS

### 2.1 Classification of major lung cell populations in a mouse model of DAD

**FIGURE 1:**
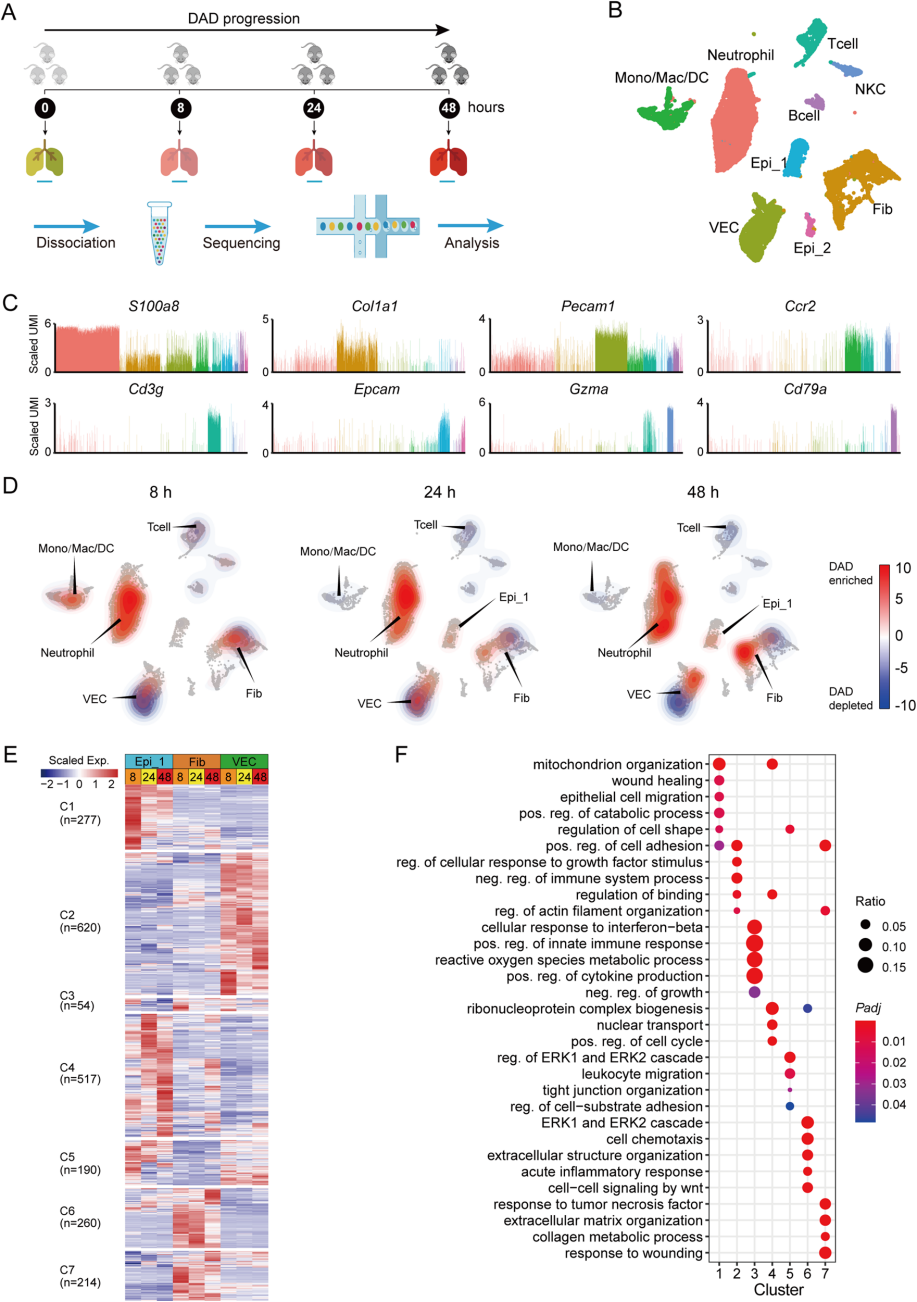
Diverse lung cell populations in a mouse model of diffuse alveolar damage (DAD) revealed by scRNA‐seq. (A) Workflow of scRNA‐seq experimental design. (B) Uniform manifold approximation and projection (UMAP) projection of 36,810 single cells obtained from the homeostatic control lung tissues (0 h) and the DAD lung tissues (8, 24, and 48 h post ricin challenge). (C) Expression patterns of canonical cell markers for identification of cell populations in UMAP plot. Two clusters, co‐expressing epithelial cell marker Epcam, are annotated as Epi_1 and Epi_2, shown in (B). The indicated values are log‐normalized UMI per cell. (D) Density plots showing abundance changes for different cell populations at 8, 24, and 48 h compared to 0 h on UMAP graph. (E) Heat map showing differentially expressed genes (DEGs) (rows) among different cell populations at 8, 24, and 48 h compared to 0 h. These genes are classified into seven modules through unsupervised *k*‐means clustering. (F) GO enrichment for DEGs shown in (E).

To investigate lung cellular responses during DAD progression, mice were challenged with aerosolized ricin to induce DAD syndrome, as described in our previous study.^[^ ^4^ ^]^ We then performed 10× genomic scRNA‐seq on the dissociated pulmonary cells at 0 (homeostatic control), 8, 24, and 48 h post‐ricin challenge, followed by integration analysis using Seurat (Figure 1A). As a result, 36,810 cells were obtained and a uniform manifold approximation and projection (UMAP) of the cell landscape was created (Figure 1B), indicating an overrepresentation of lung cells. Based on highly expressed classical genotypic markers, these lung cells were classified into nine major populations (Figure 1C): neutrophils (the predominant cell population); monocyte‐lineage cells (monocyte/macrophage/dendritic cell; Mono/Mac/DC); natural killer cells (NKC); T cells; B cells; vascular endothelial cells (VEC); fibroblasts (Fib); cluster_1 epithelial cells (Epi_1); and cluster_2 epithelial cells (Epi_2).

The changing proportions of these cell subpopulations over time were assessed (Figure 1D). A global reorganization of lung immune cell populations began 8 h post‐ricin exposure, including an increase of neutrophil and monocyte‐lineage cells, and a decrease of lymphocyte‐lineage cells, including T cells and B cells (Figure 1D). This was confirmed by fluorescence‐activated cell sorting (FACS) (Figure S1) and immunofluorescence imaging (Figure S2A,B). A gradual and sequential reorganization of neutrophils, VEC, and Epi_2 was also observed, indicating a continual change in these cells during DAD progression (Figure 1D).

The obtained cells yielded a total of 2132 differentially expressed genes (DEGs), which were classified into seven major clusters based on their gene expression patterns at 8, 24, and 48 h (Figure 1E). Gene ontology (GO) enrichment analysis indicated these seven clusters had distinct biological processes (Figure 1F). The DEGs in cluster_1 were distributed in epithelial cells (Epi_1 and Epi_2), and mainly enriched in the biological processes of wound healing, epithelial cell migration, and cell shape regulation. The DEGs in cluster_2, mainly belonging to VEC, were involved in cell adhesion and binding. The 260 fibroblast‐related DEGs in cluster_6 were significantly enriched in chemotaxis and inflammatory response. These findings demonstrate the temporal change in lung structural cells during DAD progression.

### 2.2 Fibroblasts and neutrophils as the major producers of pro‐inflammatory cytokines and chemokines during DAD progression

**FIGURE 2:**
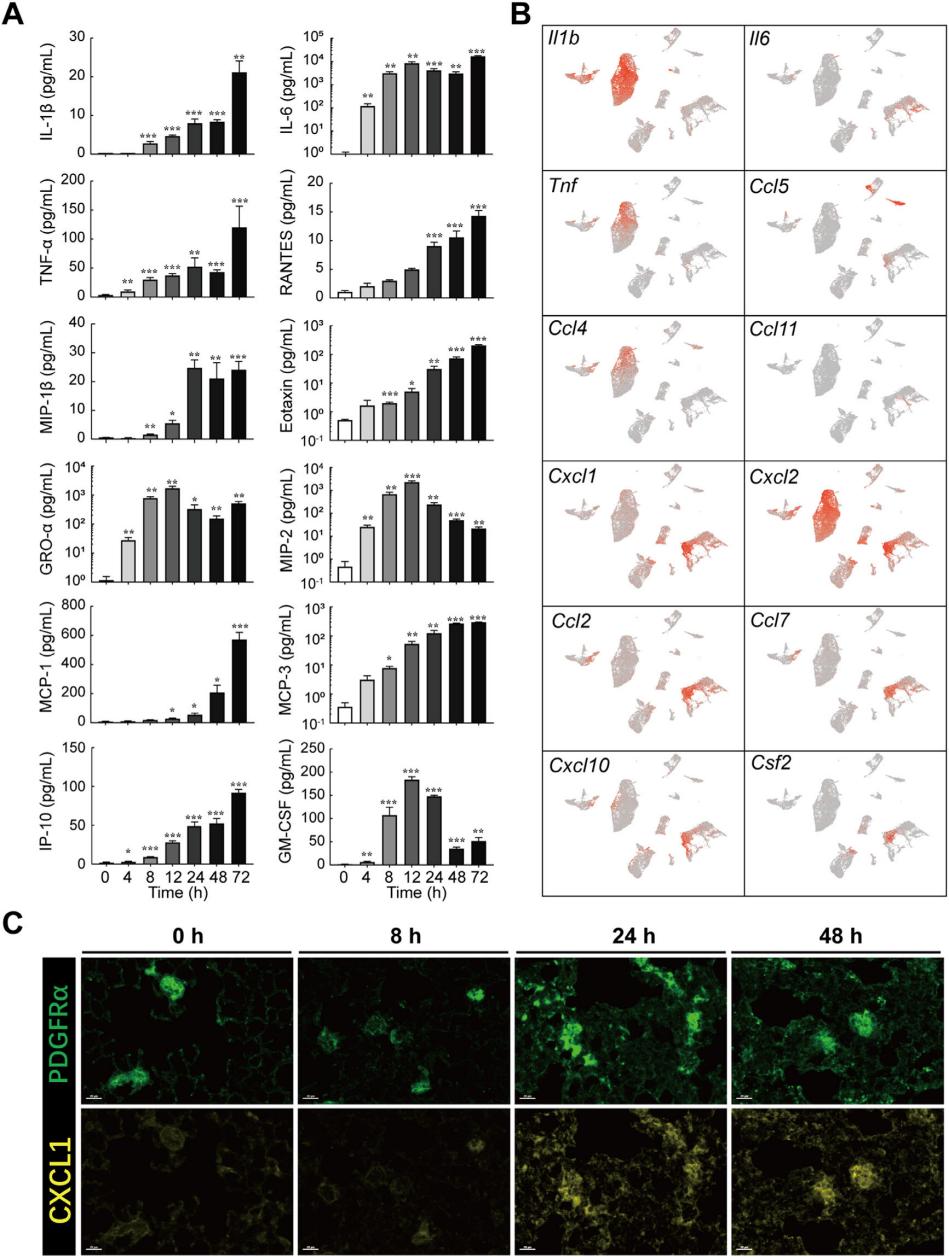
Correlation between cytokines/chemokines and cell populations. (A) Abundance of proinflammatory cytokines/chemokines in bronchoalveolar lavage fluids (BALFs) at different time points post ricin challenge. (B) Normalized intensity of cytokine/chemokine genes shown in uniform manifold approximation and projection (UMAP). (C) Immunofluorescence staining of CXCL1. Note expression in the lung tissues in PDGFRα+ fibroblast. Scale bar. *p < 0.05, **p < 0.01, and, ***p < 0.001.

The gene expression profiles of pro‐inflammatory cytokines/chemokines in bronchoalveolar lavage fluids (BALFs) at 0, 4, 8, 12, 24, 48, and 72 h post‐ricin challenge were examined with multiplex cytokine quantification assays. A total of 12 major cytokines/chemokines were highly upregulated in BALFs from 4 to 72 h post‐ricin challenge (Figure 2A), suggesting the occurrence of an inflammatory cytokine storm during DAD progression. Multiple inflammatory features were also observed in parallel histological examinations (Figure S3A,B). In detail, the abundance of IL‐6 and CXCL1 was significantly upregulated at 8 h and sustained at high levels from 8 through 48 h. GM‐CSF and CXCL2 were robustly induced at 12 h and gradually decreased at 24 and 48 h, although they remained much higher than at 0 h. The two classic proinflammatory mediators Il‐1β and TNF‐α showed a moderate increase from 4 to 48 h, and a significant increase at 72 h. Overall, ricin‐induced DAD expression patterns were characteristic of a cytokine storm, with an excess release of multiple pro‐inflammatory cytokines/chemokines, and asynchronous changes in released cytokines/chemokines denoting diverse inflammatory responses in different lung cell populations during DAD progression.

To further investigate the production sources of these cytokines/chemokines, we analyzed their gene expression profiles using UMAP plots (Figure 2B) and discrete cell populations (Figure S4A). Fibroblasts and neutrophils were the main cell populations that had higher expressed levels of these cytokines/chemokines. After ricin exposure, fibroblasts served as the major source of CXCL1, which acted as the chemoattract for mobile neutrophils (Figure 2C). We next assessed the recruitment and trafficking mechanisms of immune cells by analyzing the expression of chemokines and their corresponding receptors based on scRNA‐seq data (Figure S4B). Several potential mediators, including Cxcl1 (to Cxcr1 and Cxcr2), Cxcl9 (to Cxcr3), and Cxcl10 (to Cxcr3) exhibited high‐levels of gene expression in fibroblasts and might be involved in neutrophil recruitment.

In summary, fibroblasts and neutrophils represent the major source of multiple pro‐inflammatory cytokines/chemokines and play an important role in promoting inflammation and a cytokine storm during DAD progression.

### 2.3 Involvement of specific structural cell subpopulations in inflammatory response during DAD progression

**FIGURE 3:**
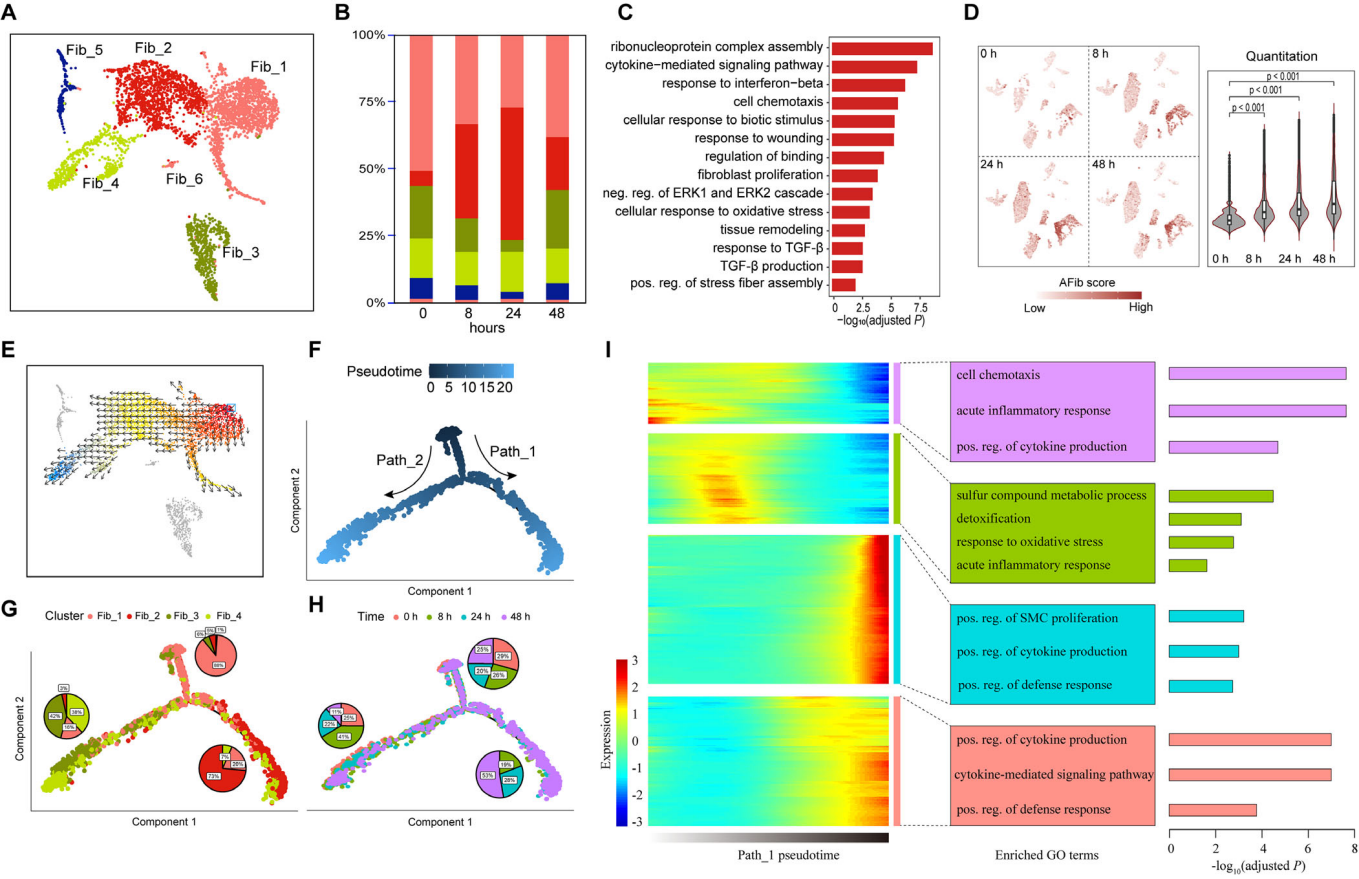
Analysis of fibroblast subpopulations. (A) Uniform manifold approximation and projection (UMAP) projection of all fibroblast cell subpopulations colored by Seurat annotated subclusters. (B) Bar plot depicting proportions of fibroblast subpopulations. (C) GO enrichment analysis of significantly upregulated genes between AFib and Fib_1. (D) AFib gene signature scores across 0, 8, 24, and 48 h. (E) Cell state transition obtained by VECTOR. (F) Two cell state transition paths inferred by Monocle software. (G) Distribution of fibroblast subpopulations in each branch of the trajectory. (H) Distribution of different experimental conditions in each branch of the trajectory. (I) Heatmap showing the dynamic changes in gene expression along the pseudotime (left panel) and the enriched biological processes in each gene module (right panel).

To further explore the role of fibroblasts in the inflammatory response, fibroblasts in the first‐level of clustering were subjected to a second‐level of unsupervised clustering. Fibroblasts could be further clustered into six subpopulations Fib_1 to Fib_6 (Figure 3A) and the cell numbers of different subpopulations were notably dynamic (Figure 3B), indicating that fibroblasts possessed highly heterogeneous subpopulations that likely had diverse functions during DAD progression. Fib_1 comprised a major proportion of fibroblasts in homeostasis, with a high abundance of homeostatic marker genes such as Mgp and Dpt, and it thus represented the homeostatic resident tissue fibroblast subpopulation (Figure 3B and Figure S5A). The proportion of Fib_2 significantly increased during the first 24 h post‐ricin exposure, followed by a gradual decrease at 48 h. Fib_2 highly expressed cytokine/chemokine genes such as Cxcl2 and Csf2 (Figure S5A), indicating an increased inflammatory response in Fib_2 during DAD progression, which was also supported by GO enrichment analysis (Figure 3C). We termed the Fib_2 subpopulation as activated fibroblast (AFib). Further analysis on time‐specific unintegrated UMAPs revealed increasingly inflammatory responses due to continuously increased AFib scores from 8 to 24 to 48 h (Figure 3D). Fib_3 showed a significant decrease during the first 24 h with a clear topographical separation from Fib_1 and AFib on the UMAP plot. Fib_4 displayed no obvious change in its ratio of cell populations during DAD progression. Fib_5 and Fib_6 were excluded in the following analysis as they represented only minor cell subpopulations (Figure 3B).

Activity scores of 14 major signaling pathways for fibroblast subpopulations were next explored using PROGENy (Figure S5B). Homeostatic Fib_1 cells were enriched in the estrogen pathway. AFib cells were enriched in inflammatory and intercellular communication signaling pathways, such as NKkB, TNFa, EGFR, and PI3K. Fib_3 cells displayed significant enrichment in hypoxia and TGFa pathways.

To study the dynamic transition of fibroblast subpopulations, we inferred state trajectories using VECTOR analysis (Figure 3E). Fib_1, AFib, and Fib_4 all showed a continuous trajectory starting from homeostatic Fib_1 to inflammatory AFib, and then to Fib_4. Further pseudotime analysis using Monocle indicated two cell state transition paths (Figure 3F,G). Fib_1 mainly resided at the starting region of the trajectory paths, while AFib and some of the Fib_4 cells were located at the far end of one trajectory Path_1. Fib_3 and the rest of the Fib_4 cells were situated at the far end of trajectory Path_2. AFib cells were predominantly distributed in ricin‐exposed tissues and not in controls (Figure 3H), indicating their potential role during DAD progression. We further investigated DEGs associated with transitional states in Path_1. Significant upregulation of many inflammatory response‐related genes (responsible for cytokine production, positive regulation of the defense response, and the cytokine‐mediated signaling pathway) occurred in the terminal state for fibroblasts (Figure 3I), indicating higher proinflammatory activity for these cells (especially AFib) during DAD progression.

The initially identified epithelial cell populations, Epi_1 and Epi_2, were further classified into six subpopulations, namely Epi_1.1 to Epi_1.3 and Epi_2.1 to Epi_2.3, under second‐level unsupervised clustering (Figure S6A). We observed a significantly increased proportion of Epi_1.1 during the first 24 h post‐ricin exposure (Figure S6B); Epi_1.1 belonged to aberrant epithelial cells with many significantly upregulated genes that were involved in stress adaptation (Csf1, Tnfaip3, and Cxcl2), adhesion (Icam1), and further triggering of early immune responses (Figure S6C). GO analysis showed significant enrichment of inflammatory response and receptor signaling pathways in Epi_1.1 (Figure S6D), suggesting a rapid epithelium‐intrinsic inflammatory response of Epi_1.1, which were then annotated as activated epithelial cells (AEpi).

The sole VEC population initially identified was re‐clustered into five subpopulations, namely VEC_1 to VEC_5, under second‐level clustering (Figure S6E). VEC_2 was the predominant subpopulation in homeostatic lungs (0 h), whereas VEC_1 was predominant at 8 and 24 h (Figure S6F). GO analysis for the DEGs in VEC_1 showed significant enrichment for cell adhesion and leukocyte migration, suggesting a promotor effect on immune reaction (Figure S6G). We termed VEC_1 as activated endothelial cell (AEndo). Tracing the gene signature of AEndo in time‐specific unintegrated UMAPs revealed increased signature scores at 8, 24, and 48 h (Figure S6H).

Collectively, AFib (Fib_2), AEpi (Epi_1.1), and AEndo (VEC_1), as the subpopulations of lung fibroblast, epithelial cell, and VEC, respectively, exhibited pro‐inflammatory gene expression profiles and together facilitated the inflammatory response during DAD progression.

### 2.4 Complex intercellular communications during DAD progression

**FIGURE 4:**
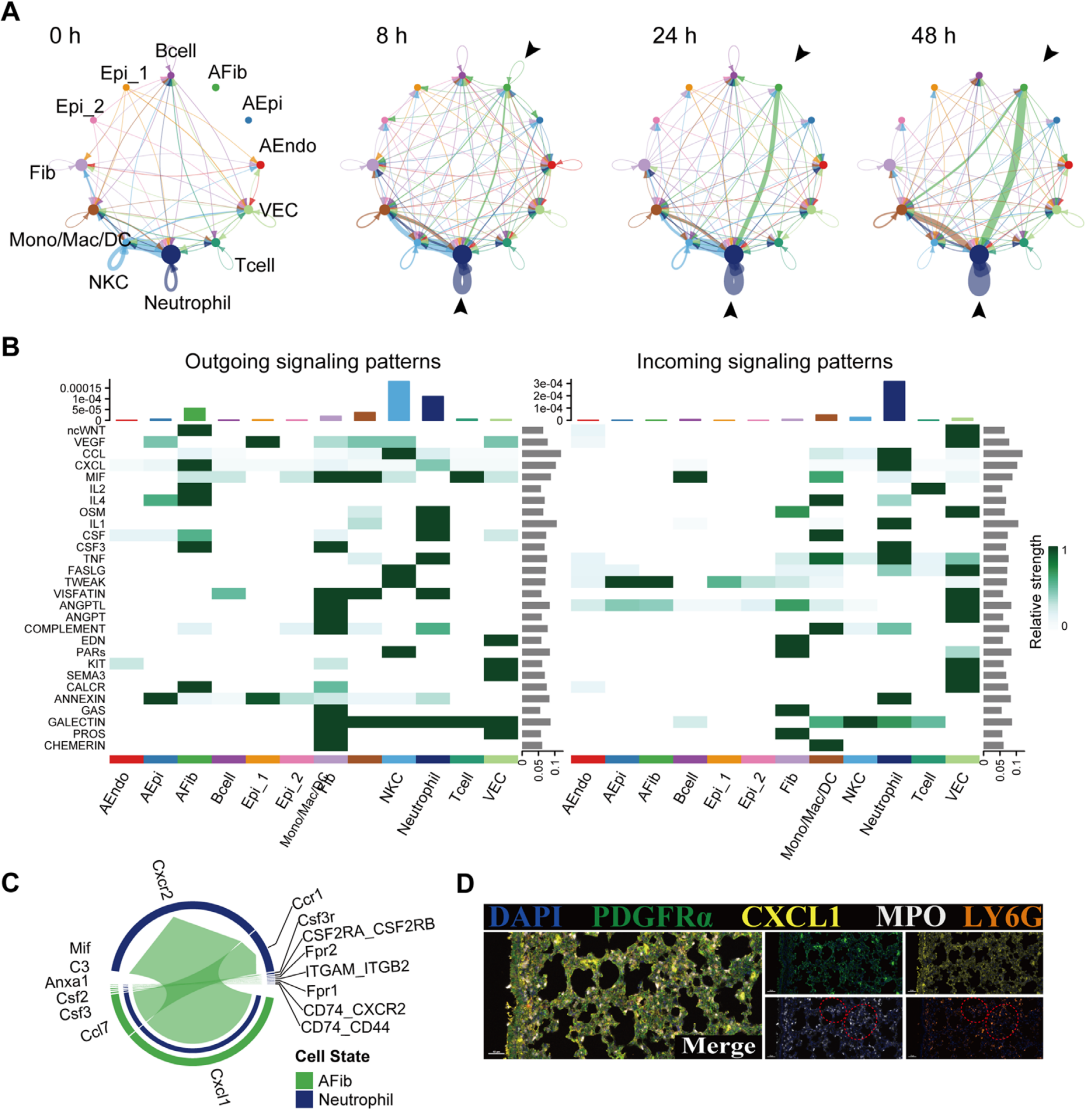
Cell–cell interactions. (A) Interaction net count plot among diverse cell populations in diffuse alveolar damage (DAD) lung tissues at 0, 8, 24, and 48 h. Two cell types with putative crosstalk were connected with line. Line color represents ligand expressed by the individual cell type. Line thickness represents the number of interactions between the two cell types. Loops represent autocrine patterns. Map quantifies potential communications of cell populations. (B) Comparison of incoming and outgoing signaling patterns for secreting and receiving cells at 24 h. (C) Contributions of LR pairs to CXCL signaling pathways from AFib to neutrophil at 24 h. (D) Representative image of multiplex immunofluorescent staining of PDGFRa, CXCL1and MPO. Note (red dashed circle) that neutrophil‐recruited chemokine (CXCL1) and neutrophil marker (MPO, LY6G) are co‐localized in the lung tissues.

To obtain the cellular interaction profile, CellChat was used to calculate interaction scores, and a weighted interaction map (Figure 4A) was generated based on the average expression levels of soluble signaling mediators and their receptors between two cell populations. Cell–cell interaction analysis revealed that AFib had an increased outgoing connection score and that neutrophils represented the main inbound receptor (Figure 4A), suggesting that AFib mediated neutrophil recruitment during DAD progression. Notably, neutrophils showed an enhanced interaction with themself, indicating an autocrine loop for neutrophil recruitment. Furthermore, the increased interaction between neutrophil and monocyte‐lineage cells across 8, 24, and 48 h indicates an enhanced interplay between these two cell populations. Additionally, NKC and neutrophils showed obvious signaling interactions at 0, 8, and 24 h, but this interaction decreased at 48 h (Figure 4A).

Within the separate crosstalk compartments, we observed several common and stage‐specific ligand‐receptor (LR) pairs (Figure 4B and Figure S7A–C). Consistent with cytokine production source analysis, AFib as the major donor showed the strongest interaction with neutrophils (the major signal receptor) and monocyte‐lineage cells at 24 and 48 h. These interactive signals were related to chemokine and cytokine signaling such as CXCL, CSF, and CALCR. In addition, we observed significantly differentiated communication in EGF/FGF/PDGF signaling pathways between AEpi and fibroblasts at 8 h (Figure S7B). Interactions between AFib and neutrophils were mainly mediated by the LR pairs Cxcl1‐Cxcr2 (Figure 4C,D), while those between AEpi and fibroblasts were mediated by the LR pairs Hbegf‐Egfr, Pdgfb‐Pdgfra, and Fgf1‐Fgfr1 (Figure S8).

Taken together, results show intercellular communication was widespread among fibroblasts, epithelial cells, and endothelial cells during DAD progression, facilitating the activation of complex inflammatory cascades. In particular, AFib and neutrophils were the major signal sender and receptor, respectively. These data suggest that AFib promotes DAD progression by mediating neutrophil recruitment.

### 2.5 Spatiotemporal architecture of inflammatory microenvironment during DAD progression

**FIGURE 5:**
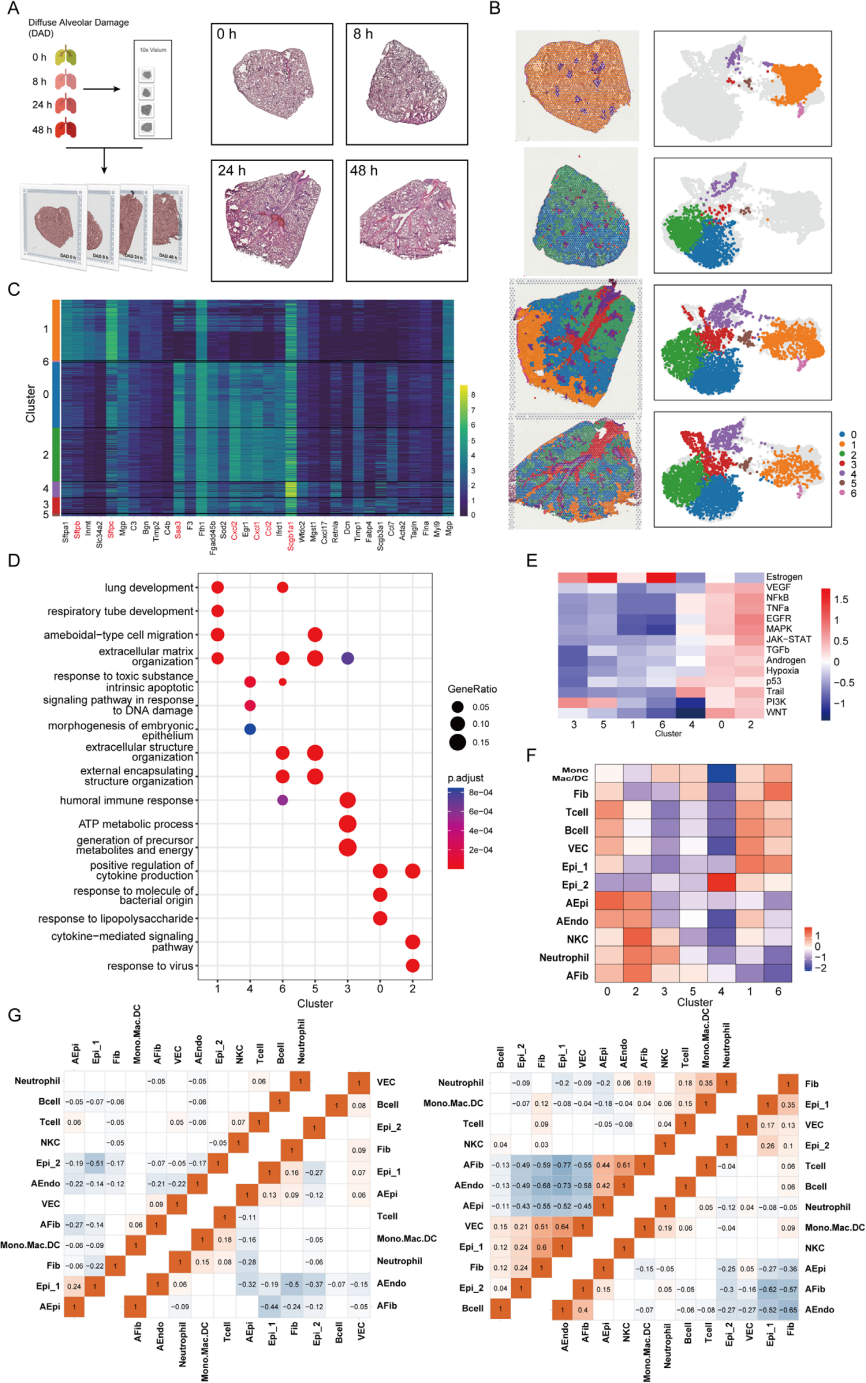
Overview of STomics analysis. (A) STomics analysis of lung tissue sections at 0, 8, 24, and 48 h. (B) Seven distinct clusters identified in the four lung tissues. The integrated data were first normalized and subjected to graph‐based clustering, and then depicted as an overlay of the spot cluster annotation across the tissue (left) and embedded in uniform manifold approximation and projection (UMAP) space (right). (C) Heatmap depicting expression values of the most variable genes among the seven clusters. Marker genes of cell populations were labeled with red color (D) GO enrichment for the genes among distinct spatial clusters. (E) Pathway activities obtained by VECTOR. (F) Expression profile comparison between STomics clusters and scRNA‐seq cell populations. (G) Heatmap of Pearson's correlation values evaluating the relationship between different inferred cell populations in STomics. Left to right: 0, 8, 24, and 48 h. See also supplementary Figure S11A,B.

To assess the spatial organization of cell populations during DAD progression, we performed STomics using a 10× Genomics Visium Spatial Gene Expression Assay for lung tissue sections at 0, 8, 24, and 48 h post‐ricin challenge (Figure 5A). A total of 10,094 tissue‐covered spots were obtained, each spot containing 3960 unique genes (median) and 14,107 unique molecular identifiers (UMIs, mean). In general, the number of UMIs in the DAD samples (8, 24, and 48 h) was higher than those in the homeostatic control samples (0 h) (Figure S9).

STomics data were first classified into different regions using principal component analysis (PCA) based on variably expressed genes among all STomics spots. After correcting for batch differences, unsupervised clustering identified seven clusters (Figure 5B). Splitting the UMAP plot showed notable condition enrichment across four time points (Figure 5B). Overall, cluster_1 was enriched in the homeostatic control samples, and cluster_0 and cluster_2 were enriched in the DAD samples, while cluster_3, cluster_4, and cluster_5 were observed in all samples. We then explored the laminar distribution of some canonical marker genes, such as Epcam, Col1a1, Pecam1, and S100a8 (a classical innate immune cell marker), identified in scRNA‐seq data; the STomics data further confirmed the fundamental remodeling of lung cell populations during DAD progression (Figure 5B,C), while the significant upregulation of neutrophil marker genes indicated that neutrophils represented the major recruited immune cells during DAD progression (Figure S10A–E). We also found it difficult to distinguish the spatial registration of overlapping cell populations in some clusters (Figure S10A–D).

GO enrichment analysis was performed to further explore the functions of different clusters identified in STomics (Figure 5D). The detected genes in cluster_1 were highly enriched in the homeostatic control samples and mainly related to basic physiological functions, such as lung development, respiratory tube development, and extracellular matrix organization. The detected genes in cluster_0 and cluster_2 were mainly enriched in inflammatory processes, including positive regulation of cytokine production and cytokine‐mediated signaling pathways. The activity score of intercellular communication, immune modulation, and proinflammatory pathways was also enhanced in cluster_0 and cluster_2 (Figure 5E). Overall, the histopathological regions in cluster_0 and cluster_2 played a vital role in inflammatory response during DAD progression, which was reminiscent of the AFib subpopulation identified in scRNA‐seq data (Figure 3A,C).

Given that each spot typically captured several overlapping cell populations, we next de‐convolved the STomics data with the signature genes from scRNA‐seq data. The analysis revealed that clusters from STomics were dominated by a subset of specific cell populations such as fibroblasts, epithelial cells, endothelial cells, and neutrophils (Figure S11A). These main cell populations were further compared between STomics and scRNA‐seq data (Figure 5F). Genes in cluster_0 showed the significantly upregulated expression of epithelial cell markers, indicating enrichment of AEpi. The highly expressed genes in cluster_1 belonged to Epi_1 and AEndo. Genes in cluster_2 displayed high expression of immune cell markers such as AFib and neutrophils, while cluster_3 contained highly expressed neutrophil gene markers. The enriched marker genes in cluster_4 were derived from Epi_2. Cluster_5 was not enriched for any cell marker genes and cluster_6 represented a minor cell population, with a mixture of several cell populations. Thus, STomics data indicated the spatiotemporal heterogeneity of cell populations across 8, 24, and 48 h, which was confirmed by the enrichment of specific cell populations from scRNA‐seq data (Figure 5F).

Spatial relationship and co‐localization among different cell populations for the STomics slices (Figure 5G, Figure S11B) were then analyzed. As expected, the activated lung structural cells AEpi, AFib, and AEndo were clustered together, suggesting the formation of an inflammatory microenvironment by these cells. We also observed co‐localization of AFib and neutrophils at 24 and 48 h (Figure 5G); this agrees with the intracellular interaction findings, that AFib represents the major source of chemoattractants for neutrophil recruitment, as shown in the scRNA‐seq data (Figure 4B–D).

### 2.6 Spatial histopathological and gene expression patterns during inflammatory response

**FIGURE 6:**
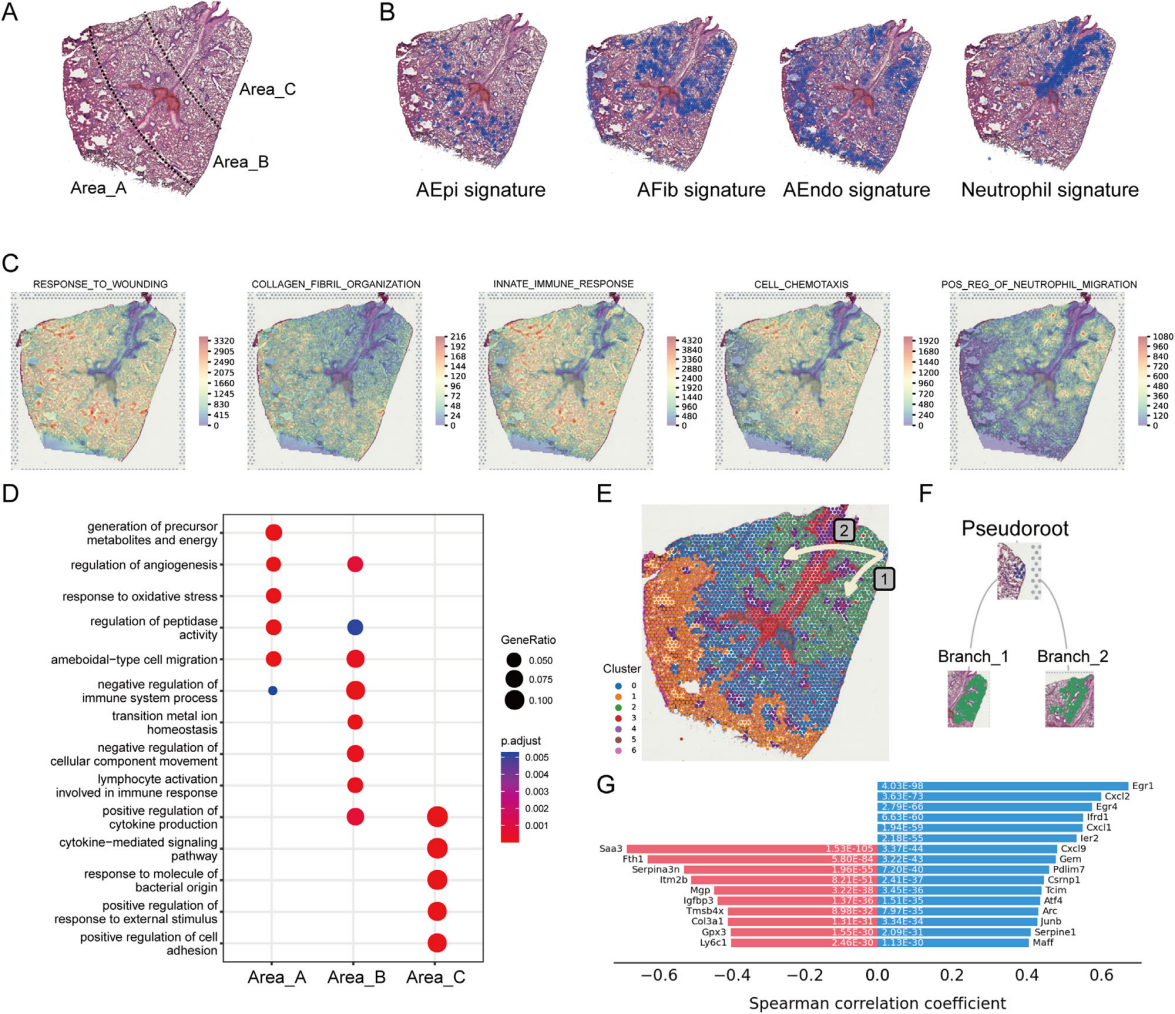
Spatial histology and gene expression patterns at 24 h. (A) Annotated brightfield images of H&E‐stained lung tissue section. (B) Visualization of AEpi, AFib, AEndo, and neutrophil signature expressions in spots of the same tissue section. (C) Expression profile of distinct functional gene modules in sots of the same tissue section. (D) GO enrichment for the marker genes in each area. (E) Visualization of cell state trajectories with tissue localization. (F) Tree plot with hierarchical layout showing the flow of progress branching from cluster_0 regions (blue) to cluster_2 regions (green). (G) Plot showing the genes with positive (blue) and negative (red) correlations with the spatial trajectory.

The spatial histopathological patterns on the STomics slice (including lung parenchyma regions in lower, middle, and upper lobe areas) at 24 h (Figure 6) were analyzed. The slice could be divided into three distinct regions based on the histopathological structures (Figure 6A): the lower lobe represented the terminal bronchial region (Area_A), the middle lobe represented the bronchiole region (area_B), and the upper lobe represented the principal bronchial region (Area_C). A collagen deposition region was distributed mostly in area_A, indicating that area_A was the main area of ricin‐induced DAD and underwent the wound healing progression. In contrast, no obvious pathological changes were observed in area_B or area_C (Figure 6A). These histopathological findings agree with the unbiased spot clustering results in Figure 5B.

The spatial enrichment profile of immune cells and activated lung structural cells in the STomics slice at 24 h (Figure 6B) was then assessed. AEpi was detected mostly in area_B, while AFib was exclusively enriched in area_C. AEndo was distributed in area_A and area_C, while neutrophil was enriched in area_C. A functional enrichment analysis revealed different biological progressions among the three areas (Figure 6C,D). Area_A was responsible for physiological reactions (such as hypoxia‐response for adaption of low oxygen content) and area_C was characterized by activated immune response and immune cell infiltration, while area_B reflected an intermediate state between area_A and area_C. Furthermore, neutrophil recruitment happened in the upper lobe areas close to the bronchi regions. Collectively, scRNA‐seq identified the major activated structural cell population AFib (Figure 3A) and STomics indicated its location in the upper lobe close to the bronchi regions (Figure 6B).

A spatial trajectory using both spatial information and gene expression profiles was then constructed. A natural progression of cluster_0 cells toward cluster_2 cells in area_C (Figure 6E,F) was observed. The root cells of the trajectory showed significant upregulation of collagen deposition markers (such as Saa3, Fth1, Mgp, and Col3a1), while the end cells of the trajectory displayed significant upregulation of transcription factors (such as Egr1, Egr4, Atf4, and Junb), chemokines (such as Cxcl2, Cxcl9), and transcriptional co‐activators (such as Ifrd1) (Figure 6G). These results are consistent with the observations from scRNA‐seq data (Figure 4A–D), again suggesting that AFib served as the major trigger for neutrophil recruitment in the upper lobe.

Based on the above combined analysis of scRNA‐seq and STomics data, we speculated that AFib boosted a rapid and robust immune response via recruitment of neutrophils, and then the recruited neutrophils produced many more proinflammatory mediators to further induce airway inflammation outbreak in the upper lobe, an area we termed “inflamed niche.”

### 2.7 Spatial intercellular interaction between AFib and neutrophil in inflamed niche

**FIGURE 7:**
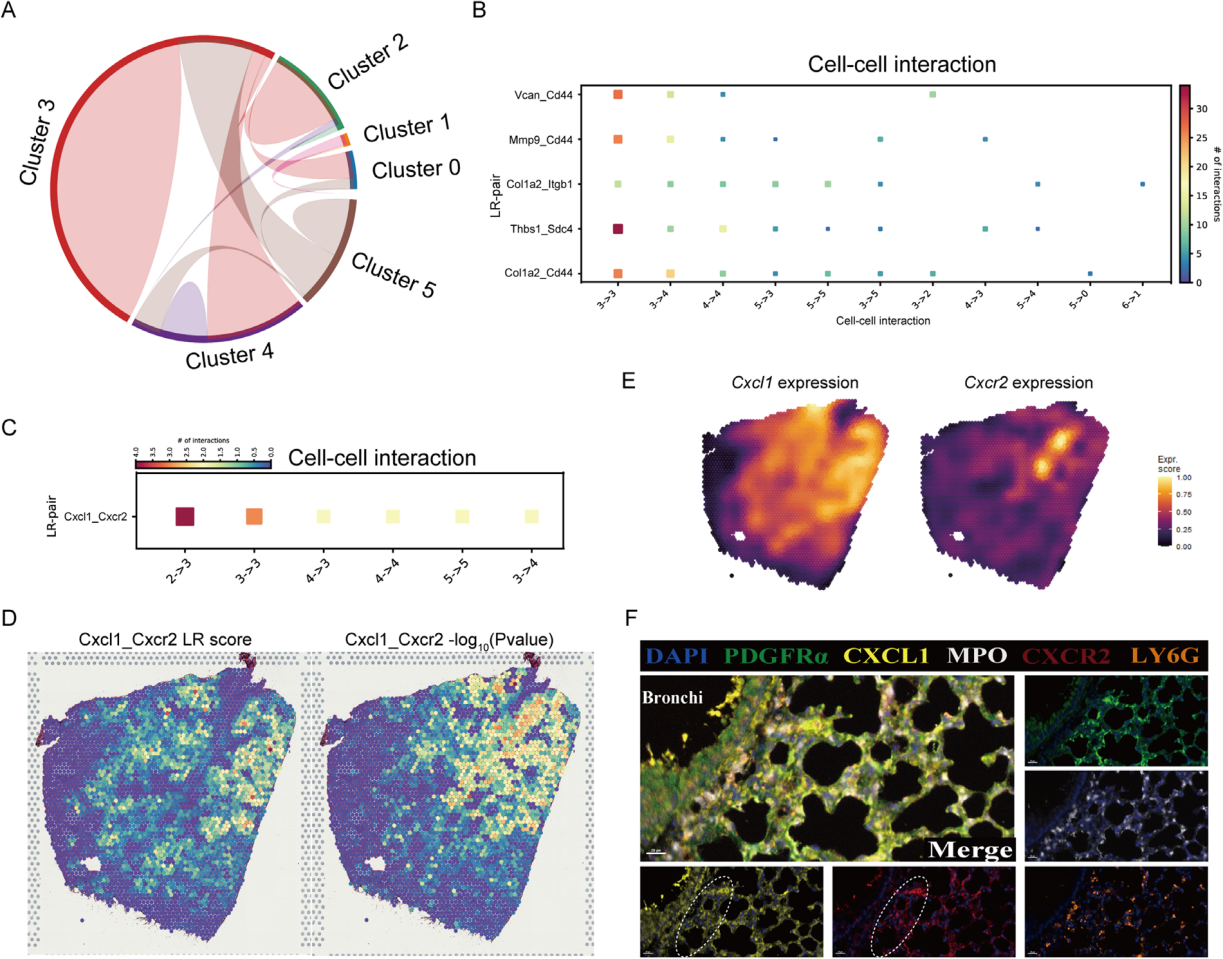
Spatial cell population interactions at 24 h. (A) Chord‐plot visualizing interactions between clusters. (B) Heatmap showing the top 5 individual cell–cell interactions across multiple LR pairs concurrently. (C) Heatmap showing the interaction weight and direction of individual spatial cluster. (D) Visualization of Cxcl1‐Cxcr2 gene pairs score and *P‐*value at spot level in the spatial context. (E) Expression pattern of Cxcl1 and Cxcr2 genes in the spatial context. (F) Representative image of multiplex immunofluorescent staining of PDGFRa, CXCL1, MPO, and CXCR2. Note (white dashed circle) that neutrophil‐recruited chemokine (CXCL1) and corresponding neutrophil receptor (CXCR2) are co‐localized around the bronchioles.

We studied the intercellular interaction between AFib and neutrophils in a spatial context (Figure 7, Figure S9) using stLearn, which can identify intensive signaling activity among different spatial regions by calculating LR co‐expression scores of cell population diversity between individual “spots.” Figure 7A shows that cluster_1 spots had fewer interactions with other areas, which might be due to the physical constraint of cluster_1 cells by collagen deposited in response to ricin‐induced injury. By contrast, cluster_3 spots showed the highest level of interaction with other regions, suggesting active intercellular communication and molecule exchange of cell populations in cluster_3. In addition, the top five active LR pairs, Col1a2‐Cd44, Thbs1‐Sdc4, Col1a2‐Itgb1, Mmp9‐Cd44, and Vcan‐Cd44, were enriched for the functions of cell migration and cell‐matrix interaction (Figure 7B) and were dominantly distributed in the inflamed niche (Figure S12). Overall, these LR pairs influenced regional or longer‐range cellular interactions and guided intercellular proximity, thereby facilitating the formation of an “inflamed niche.” Additionally, the gene pair Cxcl1‐Cxcr2 showed the highest degree of interaction and was distributed between cluster_2 and cluster_3 (Figure 7C,D), consistent with the above‐described strong interaction between AFib and neutrophils in scRNA‐seq data (Figure 4B,C). In addition, gene expression and immunofluorescent staining also suggested a mechanism whereby AFib promotes mobile neutrophil infiltration by the CXCL1‐CXCR2 chemokine axis (Figure 7E,F). Overall, a close and strong spatial intercellular interaction between hyper‐inflammatory AFib and neutrophil cells facilitated the consequence of an inflammation outbreak in the airway during DAD progression.

## 3 DISCUSSION

DAD is a complex condition characterized by hyperinflammatory responses in the lung, involving multiple interactions among resident pulmonary structural cells and infiltrating immune cells. In the present work, we used a mouse model of non‐infective DAD (induced by aerosolized intratracheal inoculation of ricin toxin) to investigate early DAD pathobiology, since this model has been shown to exert inflammatory injury (rather than inflammatory cell death) on local tissues by host immune cells.^[^ ^2a^ ^]^ Our work reveals the dynamics of histopathological changes, inflammatory cytokine profiles, and infiltrating patterns of immune cells during DAD progression in the lung, and we construct a spatiotemporal transcriptomic atlas of lung tissues at the level of cell composition and state. Our results suggest a key role of lung resident structural cells (primarily fibroblasts) in boosting neutrophil recruitment and lung inflammation. We further identify a potential role of the CXCL1‐CXCR2 chemokine axis in promoting the infiltration of neutrophils during DAD progression. These findings enable a deeper understanding of the complicated cellular and molecular mechanism for DAD pathogenesis and provide a valuable resource for developing effective therapeutic strategies against lung inflammatory diseases.

Fibroblasts are located in the interface between airway epithelial cells and vascular endothelial cells and are thus well‐placed to maintain a balance between boosting a sufficient immune response, while compromising between lung function and immunity‐induced damage.^[^ ^7^ ^]^ In this study, we observed a DAD‐responsive fibroblast subpopulation, termed AFib, with proinflammatory features in the early‐stage of DAD progression. Our multi‐dimensional data suggest that the homeostatic fibroblasts were reprogramed to an activated state (AFib), producing diverse inflammatory cytokines to promote the immune responses. AFib expressed higher levels of CXCL1, the major receptor of which was CXCR2, and was involved in recruiting mobile immune cells. Since CXCR2 was highly expressed on neutrophils, secretion of CXCL1 by AFib might be responsible for the accumulation of neutrophils, the major pathological feature of DAD. Based on spatial analysis through STomics, along with immunofluorescence imaging, we identified a hyper‐inflammatory region (termed the inflamed niche) consisting of AFib and neutrophils located in the principal bronchi region and unveiled this inflamed niche as the source for promoting infiltration of immune cells into the lung.

It is now commonly accepted that pneumocytes constitute more than just a barrier between the alveolar lumen and the underlying mesenchyme.^[^ ^8^ ^]^ As the first cells exposed to inhaled noxious substances, epithelial cells are crucial regulators for mediating the initial proinflammatory response by secreting the first wave of cytokines and chemokines.^[^ ^9^ ^]^ In our experimental model, alveolar epithelial cells underwent a phenotype switch that triggered the upregulation of several inflammatory mediators, including GM‐CSF, CXCL1, and CXCL2, during DAD progression. Our cell–cell interaction analysis revealed an interplay among injured epithelial cells and fibroblasts through several immunomodulatory signaling mediators, including heparin‐binding EGF‐like growth factor (encoded by Hbegf), platelet‐derived growth factor subunit B (Pdgfb), and fibroblast growth factor 1 (Fgf1). Since Hbegf‐Egfr, Pdgfb‐Pdgfra, and Fgf1‐Fgfr1 interactions are well‐known to play critical roles in the early inflammatory phase of rapidly progressive disease,^[^ ^10^ ^]^ we speculated that the hormone release upon epithelial cell death and the subsequent activation of receptors on lung resident cells would result in the initiation of immune responses. Collectively, our data pinpoint a conceptual model in which lung fibroblasts could potentially integrate damage signals emitted from injured epithelial cells, thereby executing a role in promoting neutrophil infiltration by producing inflammatory cytokines in early‐stage DAD. Therefore, neutralizing these paracrine signaling mediators might have therapeutic benefit by reversing the inflammatory fibroblast to a healthy state, further inhibiting the pro‐inflammatory effects and progression of DAD.

An increasing number of reports have shown that a dysregulated hyperinflammatory response is initiated by damage‐associated molecular patterns (DAMPs) released by damaged alveolar epithelial cells or recruited immune cells during DAD progression.^[^ ^11^ ^]^ Our previous study also indicated that Retro‐2 (a compound for protecting cells from death) reduced the expression levels of cytokines and increased the viability of cells.^[^ ^12^ ^]^ These results suggest that DAMPs play a role in initiating immune response and have potential as an intervention target in DAD. By analyzing DAD‐related cytokine storms and immune cell infiltration, the present study indicates a central role of fibroblasts for lung immune response during DAD progression through the recruitment of circulation neutrophils. We hypothesized that DAMPs released from stressed epithelial cells might drive the fibroblast activation under the complex inflammatory situation; this needs to be tested in future studies since DAMPs’ profiles were not included in our omics data.

This study has several limitations. First, only four representative stages during DAD progression (up to 48 h after injury) were monitored using scRNA‐seq and STomics. Although the integrative analysis with other research validates our findings to some extent,^[^ ^13^ ^]^ data presented here may not reflect the overall cellular and molecular dynamics during DAD pathogenesis. Additionally, each group of samples includes three murine individuals, and the single‐cell digestive juices from the three mouse lung tissues were pooled together immediately to minimize changes in gene expression in cells, but this may also obscure some heterogeneity. In addition, some data (such as the cellular distribution of inflammatory cytokines) cannot be statistically analyzed (only represented by means). Nevertheless, data presented herein, especially the phenotypic shifting of lung fibroblasts, are pertinent for DAD immunopathology, and may assist our understanding of inflammatory lung diseases.

## 4 CONCLUSION

In summary, we present a temporal‐spatial view of ongoing pulmonary immune responses in DAD lungs. This investigation suggests a key role for AFib in mediating neutrophil infiltration through integration and secretion of soluble signaling mediators, thus boosting neutrophilic lung inflammation. These findings not only provide novel insight into the pathogenesis of DAD disease, but also highlight mesenchymal subpopulations as potential therapeutic targets in the context of lung injury‐related diseases.

## 5 EXPERIMENTAL SECTION

### 5.1 Murine model and pulmonary single‐cell preparation

C57BL/6c mice (Mus musculus), used in our experiments, were purchased from Beijing Charles River Laboratory. Mice were housed under a standard diet ad libitum and maintained on a 12‐h light/dark cycle. The mouse model of DAD was generated as previously described.^[^ ^4^ ^]^ Briefly, following anesthetization with pentobarbital, aerosolized ricin (twice the half‐maximal lethal dose, approximately 0.01 mg kg^−1^) in 50 μL phosphate‐buffered saline was delivered to the mice intratracheally using a MicroSprayer aerosolizer. Control mice were treated with an identical volume of phosphate‐buffered saline. Mice were sacrificed by excessive anesthesia at the indicated time‐points after exposure. Three female mice aged 10 weeks were used for pulmonary single‐cell preparations for scRNA‐seq and flow cytometry analysis by tissue mincing and enzyme digestion. The lungs were isolated and the lobes were minced. Minced tissue was digested with collagenase A and DNase I, filtered, and resuspended in phosphate‐buffered saline. The lung cells of three mice were pooled.

### 5.2 scRNA‐seq library preparation and sequencing

Samples were loaded onto a Chromium Controller (10× Genomics, Pleasanton, CA, USA) to generate gel beads in emulsions of approximately 10,000 cells. RNAs from the barcoded cells were reverse‐transcribed and sequencing libraries were constructed with reagents from a Chromium Single Cell 3′ v3 reagent kit (10× Genomics) according to the manufacturer's instructions. Libraries were sequenced on an Illumina instrument (NovaSeq PE150; San Diego, CA, USA) aiming for an average of 100,000 read pairs per cell.

### 5.3 scRNA‐seq data preprocessing

Raw scRNA‐seq reads were processed by Cell Ranger (version 3.1.0). Reads were mapped to the genome using STAR, and downstream reads were used to generate normalized aggregate data across samples and create a matrix of gene counts versus cells. Genes expressed in fewer than three cells and cells detected in fewer than 200 genes were excluded. Low‐quality cells where > 5% of the counts were derived from mitochondrial genes were also excluded. Finally, normalized gene count was obtained through the normalization on library size.

### 5.4 scRNA‐seq data analysis

The Seurat^[^ ^14^ ^]^ R package (version 3.1.1) was used to calculate the first 2000 genes with the highest differential expressions by using the FindVariableFeatures function, PCA was employed to perform dimensionality reduction after regression on confounding factors, including percent of mitochondrial RNA and cell cycle. Findcluster function was used to calculate the cell clusters with a resolution 1.0 for major cell types and 0.2 for specific sub‐cluster. Clustering was visualized by UMAP in two‐dimensional space.

DEGs in each cluster were identified by FindaAllMarkers functions in the Seurat R package (use default parameters). Genes were considered differentially expressed with a log2 average differential expression of 0.5 and when *P* < 0.05. Canonical marker genes of known cell populations were used to annotate each cluster. Cells from epithelial, endothelial, and fibroblast compartments were subset for further analysis. Cell nomenclature for sub‐cluster was designated by functional property.

### 5.5 GO enrichment and pathway activity analysis

GO analyses were carried out by using the “clusterProfiler” R package.^[^ ^15^ ^]^ Package “org.Mm.eg.db” was used to map gene identifiers. *P*‐value was adjusted by using Benjamini‐Hochberg correction. GO terms represent biological processes.

PROGENy (V1.17.3) R package was used to estimate the activity of relevant signaling pathways.^[^ ^16^ ^]^

### 5.6 Inference of developmental directions for single cells using VECTOR

VECTOR (V0.0.4) R package was used to identify the starting cells and infers the vectors of developmental directions for cells in UMAP.^[^ ^17^ ^]^

### 5.7 Cell state transition trajectory

Monocle (V2.14) packages were used to construct trajectories to discover the fibroblast transitions.^[^ ^18^ ^]^ The identified DEGs in Seurat were used to obtain cells in pseudo‐times. “DDRtree” and “plot_complex_cell_trajectory” functions were applied to reduce dimensions and plot minimum spanning tree, respectively. Then we used “BEAM” function to analyze the DEGs for each transited path, which were shown as heatmap.

### 5.8 Cell–cell communication analysis

CellChat (version 0.5) was used to perform the cell–cell communication analysis.^[^ ^19^ ^]^ Briefly, cell annotated labels and the normalized gene expression level generated through Seurat workflow was integrated as input for CellChat. The expression level of ligand and receptor genes in each cell population was projected into the protein–protein interaction network. Permutation testing of randomized network connections was used to determine significant source‐target network connections.

### 5.9 Measurement of cytokines levels in BALF

Mouse BALF was used to measure the levels of inflammatory cytokines, including IL6, IL‐1β, GM‐CSF, TNF‐α, Eotaxin (CCL11), MIP‐1 (CCL2), MIP‐1β (CCL4), MIP‐2 (CXCL2), MCP‐3 (CCL7), IP‐10 (CXCL10), RANTES (CCL5) and GRO‐α (CXCL1). These cytokines were measured using Luminex multiple assay according to the manufacturer's protocol.

### 5.10 Antibodies and immunofluorescence stainings

For multiplex immunofluorescence staining, we followed the Opal protocol staining method for the following markers: For the colocalization assessment between fibroblast and neutrophil, Ly6G(ab238132, Abcam, 1:200) were labeled with Akoya Opal fluorophores 620; PDGFRα (ab134123, Abcam, 1:250) were labeled with Akoya Opal fluorophores 520; CXCL1 (PA5‐86508, Invitrogen, 1:50) were labeled with Akoya Opal fluorophores 570; CXCR2 (MAB2164, R&D Systems, 1:100) were labeled with Akoya Opal fluorophores 690; MPO (ab208670, Abcam, 1:200) were labeled with Akoya Opal fluorophores 780; The nucleus was labeled with DAPI (1:100, Akoya). For immune cell quantitative research: MPO (ab208670, Abcam, 1:200) were labeled with Akoya Opal fluorophores 690; Ly6G (ab238132, Abcam, 1:200) were labeled with Akoya Opal fluorophores 620; F4/80 (70076S, CST, 1:200) were labeled with Akoya Opal fluorophores 570; CD3 (ab16669, Abcam, 1:150) were labeled with Akoya Opal fluorophores 520; CD19 (ab245235, Abcam, 1:300) were labeled with Akoya Opal fluorophores 480; Ly6C (ab15627, Abcam, 1:300) were labeled with Akoya Opal fluorophores 780; The nucleus was labeled with DAPI(1:100, Akoya). All sections were cover‐slipped using Anti‐Fade Fluorescence Mounting Medium (ab104135, Abcam).

### 5.11 FACS

After the cells were counted with a Countess II FL automated counter (Thermo Fisher Scientific, Waltham, MA, USA), approximately 2 × 10^6^ cells per mouse were incubated in blocking solution containing 0.25% FcBlock (catalog number 553141, BD Biosciences, San Jose, CA, USA) in phosphate‐buffered saline for 15 min and then stained with fluorochrome conjugating antibodies for 30 min at 4°C. After staining, cells were washed and fixed with 1% paraformaldehyde in phosphate‐buffered saline for 30 min, then washed and resuspended in phosphate‐buffered saline with 0.5% bovine serum albumin. Flow cytometry was conducted with a BD FACSymphony A5 flow cytometer using BD FACSDiva software (BD Biosciences). The following antibodies were used: Fixable viability stain 510 (mouse, BD Biosciences, 564406), anti‐CD45 (mouse, BD Biosciences, 564279), anti‐CD16 (mouse, BD Biosciences, 553141), anti‐Ly6c (mouse, BioLegend, 128011; San Diego, CA, USA), anti‐Ly6g (mouse, BioLegend, 127641), anti‐CD11b (mouse, BioLegend, 101237), anti‐CD11c (mouse, BioLegend, 117308), anti‐CD64 (mouse, BioLegend, 139305), anti‐CD24 (mouse, BioLegend, 101825), and anti‐MERTK (mouse, eBioscience, 25‐5751‐80; San Diego, CA, USA). Data were analyzed by FlowJo version 10 (Ashland, OR, USA). All experiments were performed in four replicates.

### 5.12 Hematoxylin and eosin (H&E) staining

In a separate series of experiments, ricin‐injured mice and control mice were sacrificed at each measurement time point, and lungs were inflated with 4% paraformaldehyde and removed for paraffin embedding. Slices of 4‐μm thickness were stained with hematoxylin and eosin and observed under a light microscope. Semiquantitative evaluation of lung injury based on the method reported previously.^[^ ^20^ ^]^

### 5.13 Sample preparation for 10× Visium STomics

Mouse model of DAD was constructed according to the method mentioned above. Sampling time points of STomics experiments were in accord with scRNA‐seq. After resection, tissue sections from fresh frozen mouse lungs and then tissue optimization experiment was conducted as the pipeline from 10× Genomics. First, tissue staining & imaging was performed, then tissue permeabilization, fluorescent cDNA synthesis, and RNA quality assessment were carried out. Next, the tissues were removed slide imaging was performed. Finally, spatially localized gene expresison was performed (10× Genomics) following the manufacturer's instructions.

### 5.14 10× Visium STomics library preparation and sequencing

Tissue sections were first stained and imaged. Then tissues permeabilization within 30 min results in maximum fluorescence signal. Next, reverse transcription, second‐strand synthesis, cDNA amplification, quality control, library construct, and sequencing were performed sequentially, following the manufacturer's instructions. Overall, tissue sections from control to 48 h covered a total of 1795, 1832, 3389, and 3089 spots on the capture area, respectively. Sequencing data were then mapped to the mouse reference genome using the Space Ranger to derive a feature spot‐barcode expression matrix for downstream analysis.

The output files including raw UMI count matrix and image information, were processed with R and Python language. PCA was used to reduce the number of dimensions. The Louvain clustering algorithm was used to do the clustering, and the UMAP was used to show the clusters. Over H&E images, clusters and individual subpopulation distributions were viewed in a spatial context.

### 5.15 Defining cell state scores

With default settings using Seurat AddModuleScore function, we calculated cell scores to assess the level of expression of a particular predetermined expression gene set in individual cells or areas. The average expression of the genes from the preset gene set in each individual cell or spot was used to calculate the cell scores.

### 5.16 Deconvolution analysis of individual spots across slices

SPOTlight software was used to perform the deconvolution analysis of individual spots.^[^ ^21^ ^]^ It could identify the topic profile signatures by means of an NMFreg model, which were determined by optimizing the cell population proportions in the mixture of individual spots.

### 5.17 Spatial co‐localization of between cell populations

To identify spatially co‐localizing cell population pairs, we tested all putative pairs from our deconvolution analysis. For each two cell‐type pair, we calculated the Pearson's correlation values to evaluate the relationship between different inferred cell populations in Stomics. *P*‐value of < 0.05 and a positive coefficient were considered as spatial co‐localization.

### 5.18 Spatial trajectory inference and cell–cell interactions

stLearn (V0.4.0) was used to comprehensively analyze STomics data for the reconstruction of cell population evolution within a tissue.^[^ ^22^ ^]^ In detail, stLearn reconstructed spatial transition gradients within and between clusters that were connected locally by a directed minimum spanning tree optimization approach. Spatial information and gene expression profiles were integrated to identify locations in the tissue where there is high LR interaction activity. These areas were predicted to be hotspots where cell–cell interactions were more likely to occur.

### 5.19 Statistical analysis

Data were analyzed by R (version 3.6.3) and expressed as mean ± SEM. The statistical test performed was reported in each figure legend. *T*‐tests were two‐sided. *P*‐value was adjusted using Benjamini‐Hochberg correction.

## 6 CODE AND DATA ACCESSION

The scRNA‐seq and 10× Visium datasets in this study are available at Gene Expression Omnibus database (accession number GSE161524). There are no custom codes for data analysis, analysis codes used in the current study are available from the corresponding author (Dr. Dongsheng Zhou) upon reasonable request and through collaborative investigations.

## AUTHOR CONTRIBUTIONS

Duo Su, Zhouguang Jiao, Fei Chen, Huiying Yang, and Dongsheng Zhou designed the research, analyzed the results, and wrote the manuscript. Sha Li, Liya Yue, Cuidan Li, Mengyun Deng, Lingfei Hu, Lupeng Dai, Jinglin Wang, and Hanchen Zhang involved in the sample preparation, detection, and data analysis. Bo Gao and Haihua Xiao commented on the draft.

## CONFLICT OF INTEREST STATEMENT

The authors declare no conflicts of interest.

## ETHICS STATEMENT

All of the animal experiments were performed with the permission of the Institutional Animal Care and Use Committee of Beijing Institute of Microbiology and Epidemiology (approval number: IACUC‐AMMS‐13‐2017‐031).

## Supporting information

Supporting Information
