# Supplementary material for: Spatiotemporal single‐cell transcriptomic profiling reveals inflammatory cell states in a mouse model of diffuse alveolar damage
Source: Exploration (Beijing). 2023 Apr 10;3(3):20220171. doi: 10.1002/EXP.20220171 (PMC10624389; doi:10.1002/EXP.20220171)
Supplement: Supplementary file 1 — Supporting Information [file EXP2-3-20220171-s001.pdf]

Supporting Information

Spatiotemporal single-cell transcriptomic profiling reveals inflammatory cell states in a mouse model of diffuse alveolar damage

Duo Su, Zhouguang Jiao, Sha Li, Liya Yue, Cuidan Li, Mengyun Deng, Lingfei Hu, Lupeng Dai, Bo Gao, Jinglin Wang, Hanchen Zhang, Haihua Xiao, Fei Chen, Huiying Yang*, Dongsheng Zhou**

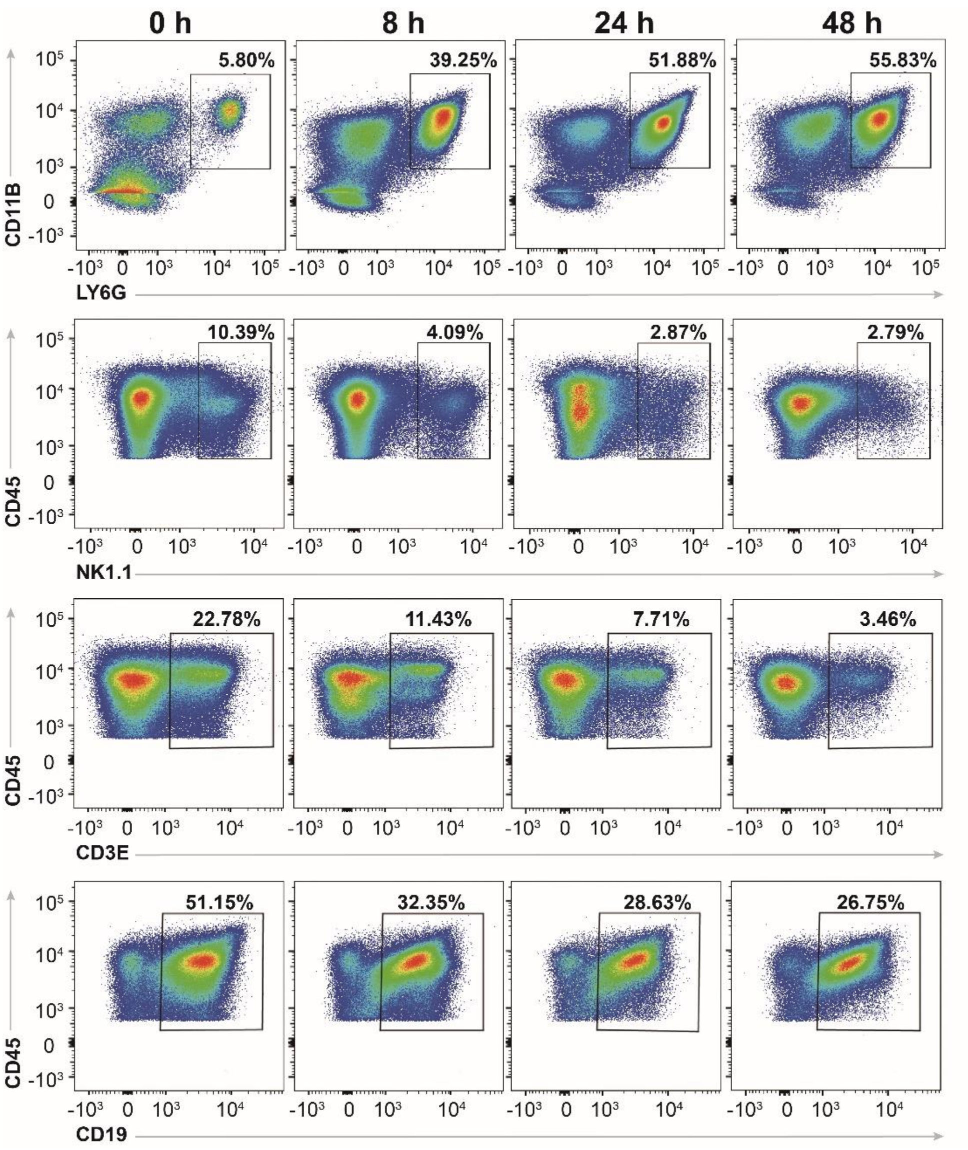

Figure S1 Representative contour plots showing the changes of cell abundance and distribution in flow cytometry during DAD progression.

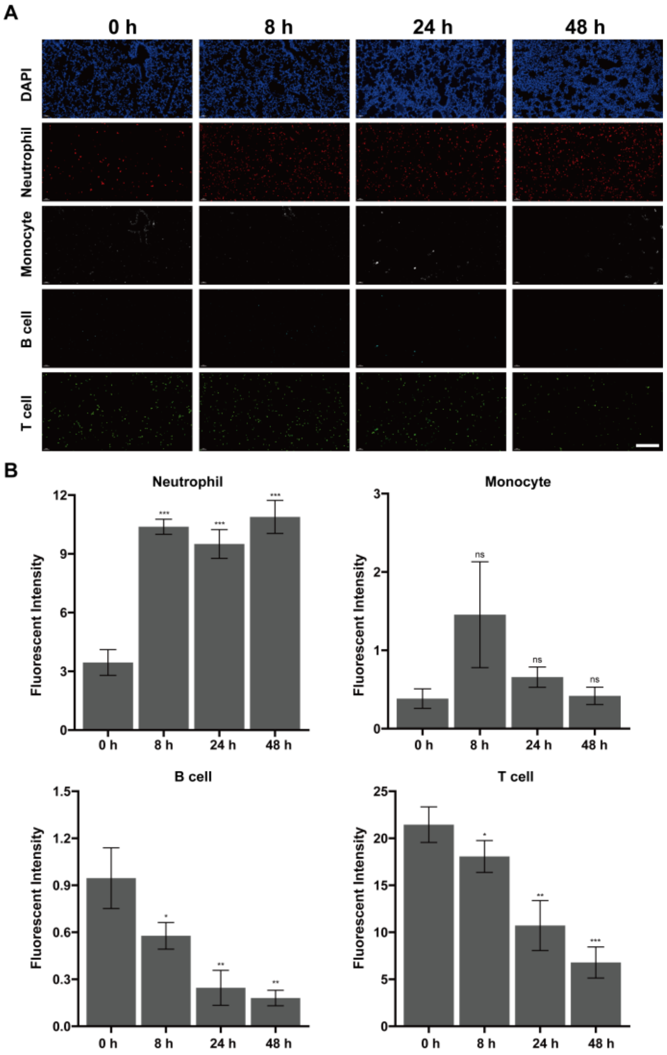

Figure S2 Immunofluorescence staining images and quantitative analysis of immune cells at different time points post ricin challenge. (A) Representative images showing the immunofluorescence staining of neutrophil, monocyte, B cells, and T cells. (B) Quantitative analysis of fluorescent intensity. * $P<0.05$, ** $P<0.01$ and *** $P<0.001$. Abbreviations: ns: not significant.

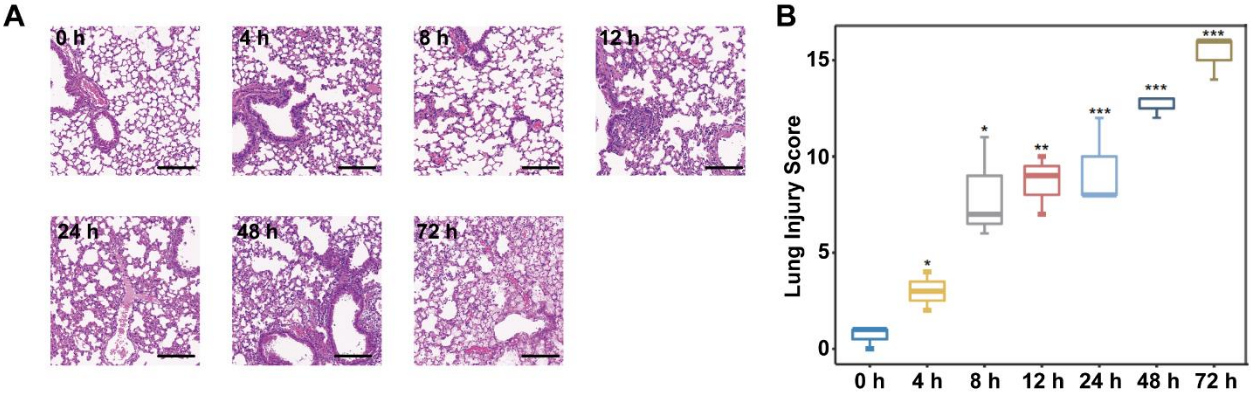

Figure S3 Histological assessment showing increased inflammation responses during SLI development. (**A**) Representative lung histopathologic images showing the ricin-exposure induced pulmonary inflammation. (**B**) Assessment of histology scores [from 0 (no damage) to 20 (maximum damage)] according to the criteria described in the Methods.

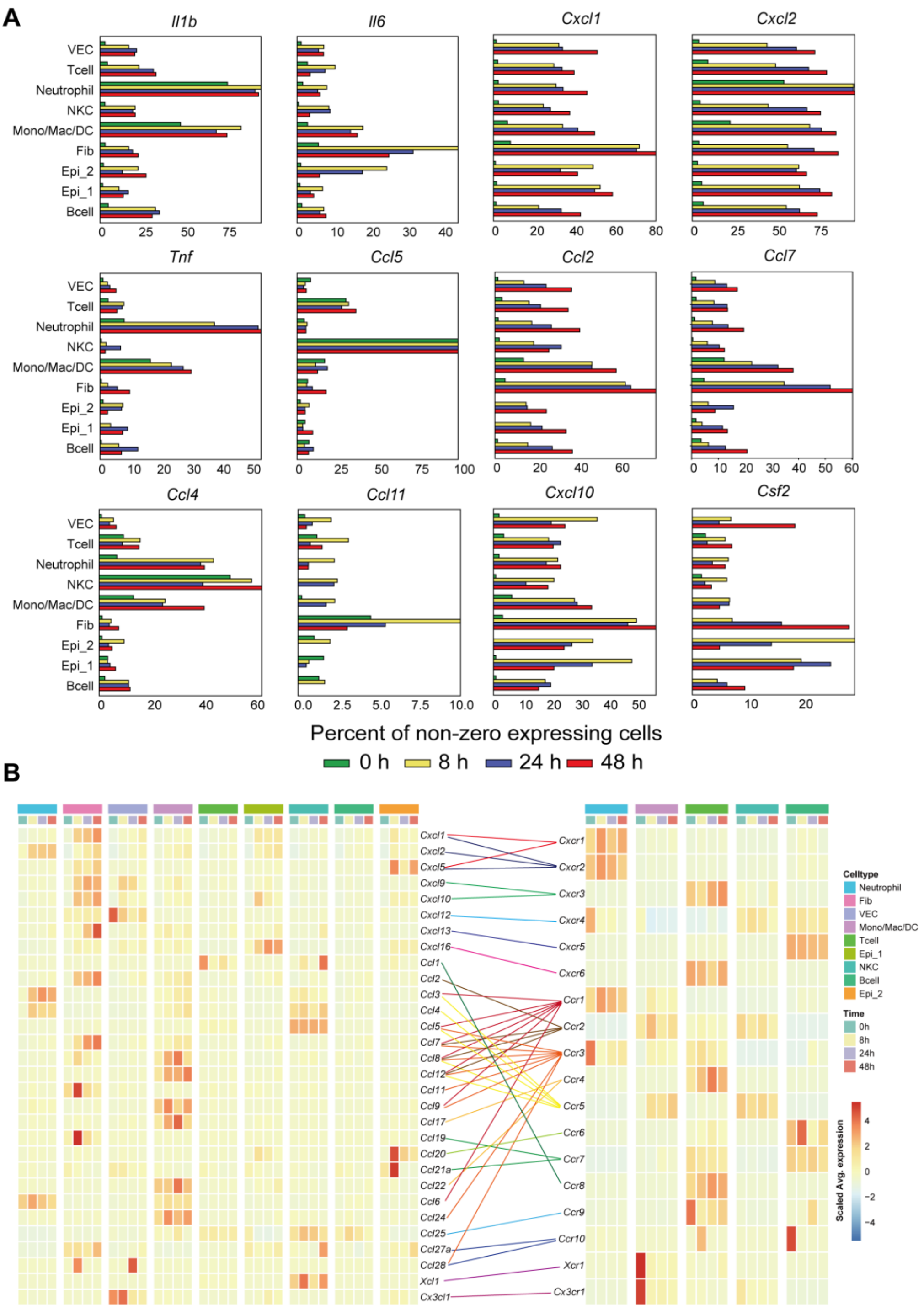

Figure S4 Distribution of cytokine/chemokine genes within cell populations at different time points post ricin challenge. (**A**) Percent of cells expressing cytokine/chemokine responsive genes. (**B**) Matching ligand and chemokine receptor pairs. Left: expression heatmap of chemokine genes across various cell types from scRNA-seq data. Right: expression heatmap of chemokine receptor genes across various lymphocyte cell types from scRNA-seq data. Matching ligands and chemokine receptors are connected with colored lines.

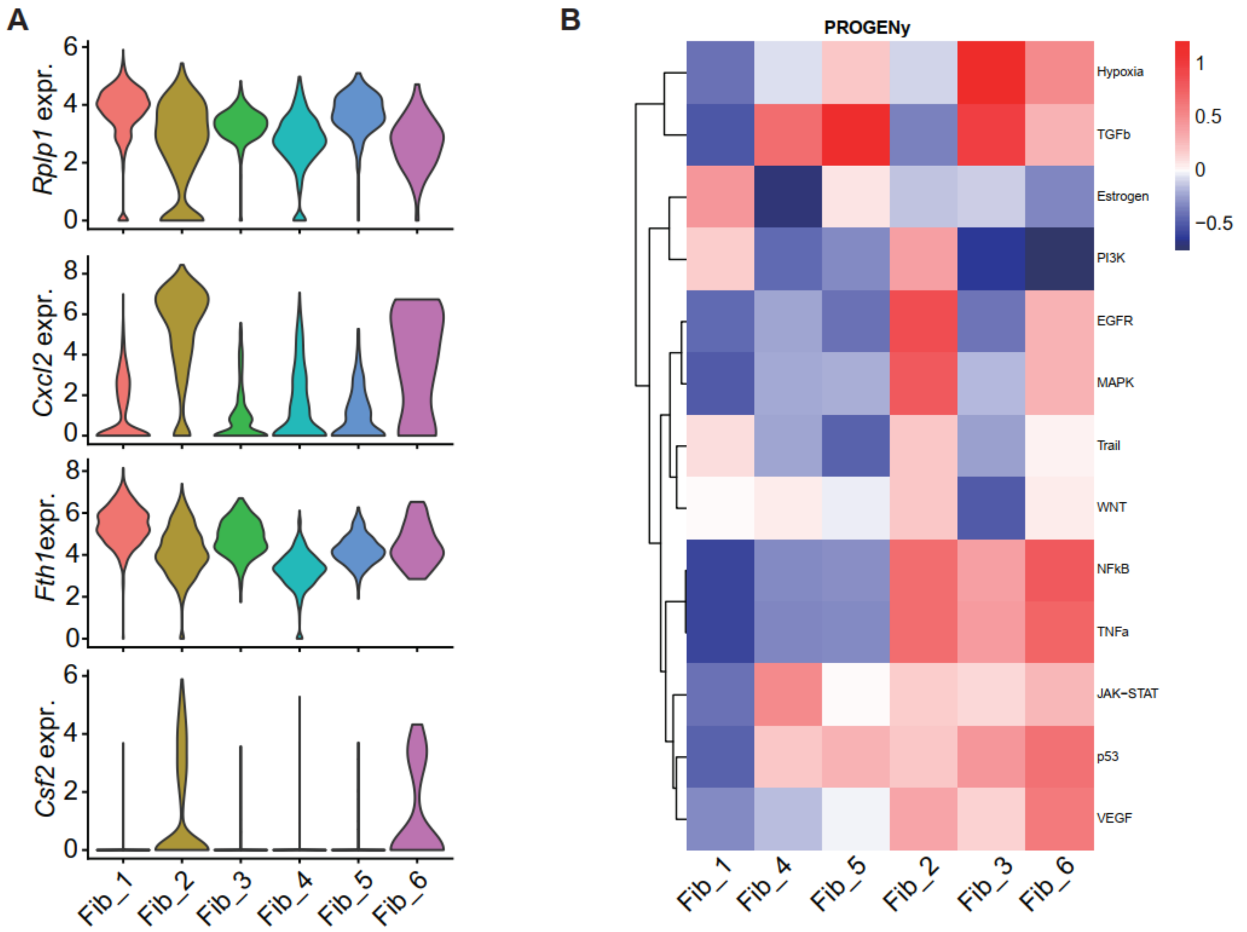

Figure S5 Significant altered DEGs (A) and corresponding enriched signaling pathways (B) of all fibroblast subsets.

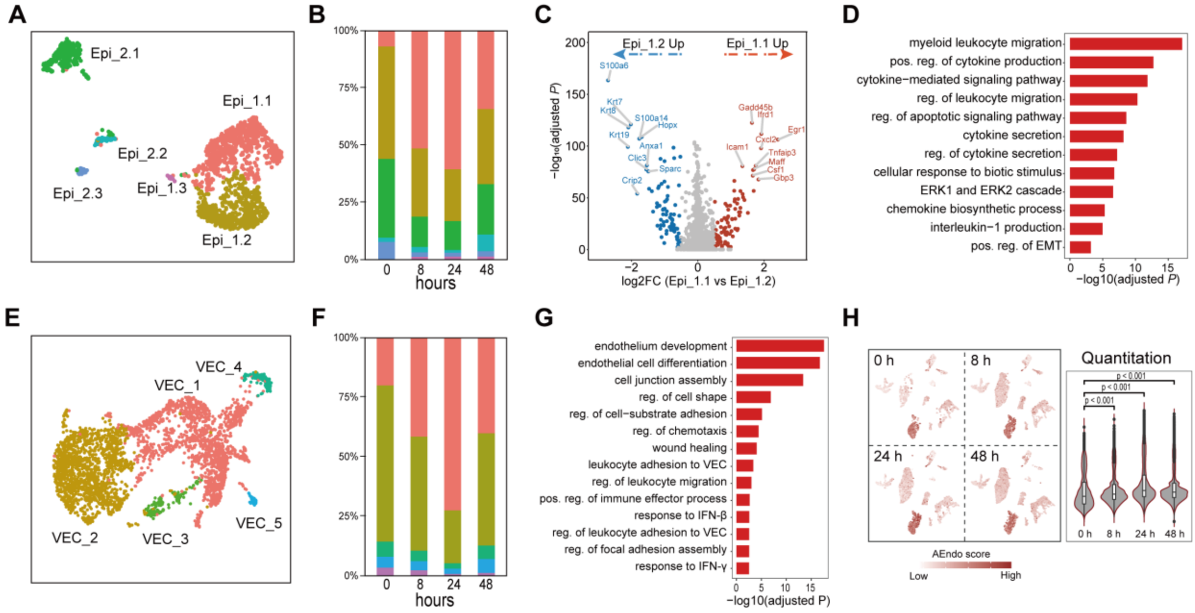

Figure S6 Reference-based cell subtype annotations revealing disease-associated perturbations in epithelial and endothelial cells. (**A**) UMAP projection of all epithelial cell subtypes colored by Seurat annotated subclusters. (**B**) The fraction of different epithelial cell subclusters. (**C**) Volcano plot showing differentially expressed genes between Epi_1.1 and Epi_1.2 cells through two-sided Wilcoxon Rank-Sum test. Red and blue dots represent significantly differentially upregulated and downregulated genes, respectively: |log2 fold change| > 0.5 and adjust p-value < 0.05. (**D**) GO enrichment analysis of the upregulated DEGs shown in (**C**). (**E**) UMAP projection of all vascular endothelial cell subtypes colored by Seurat annotated subclusters. (**F**) Bar plot depicting proportions of endothelial subclusters. (**G**) GO enrichment analysis of the upregulated DEGs between VEC_1 and VEC_2. (**H**) AEndo gene signature scores along the DAD timeline.

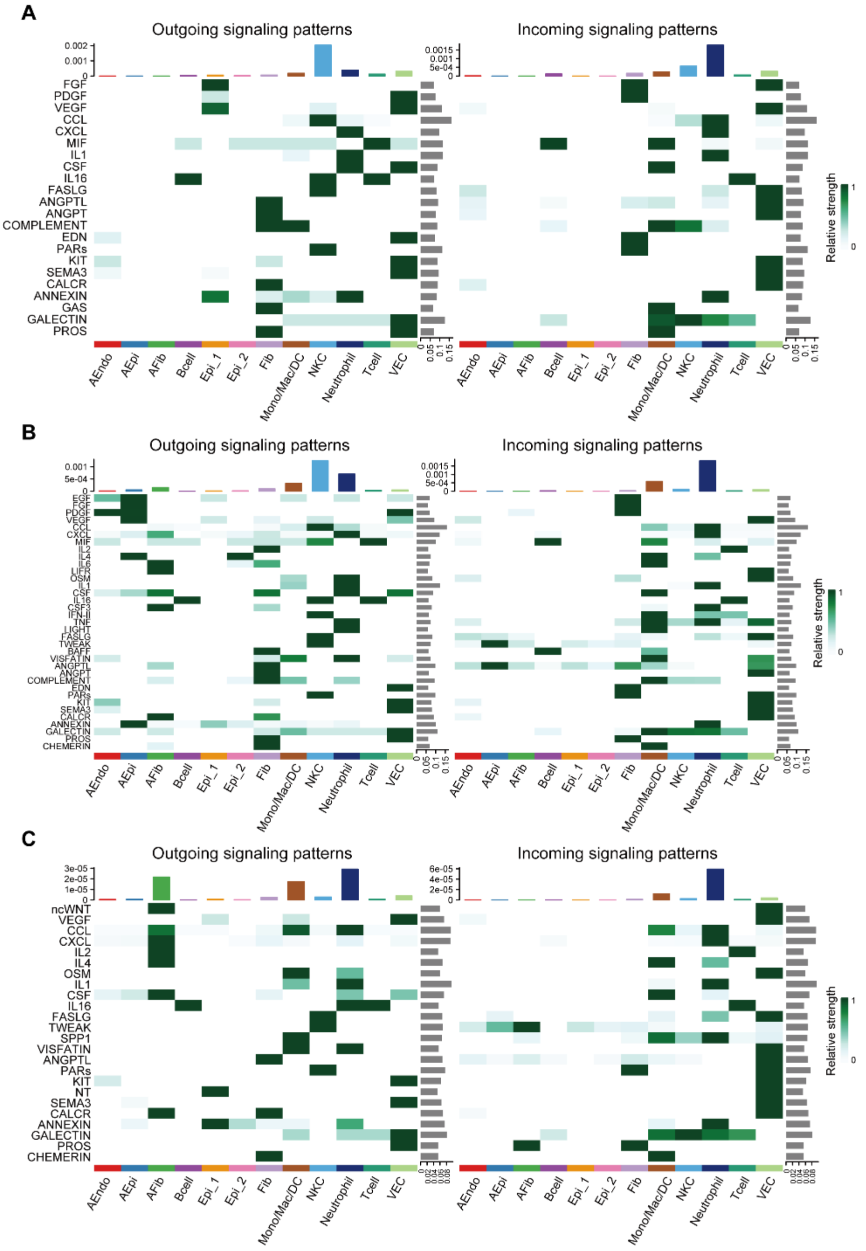

Figure S7 Intercellular signaling patterns of secreting and receiving cells at 0 h (A), 8 h (B) and 48 h (C) after ricin-exposure.

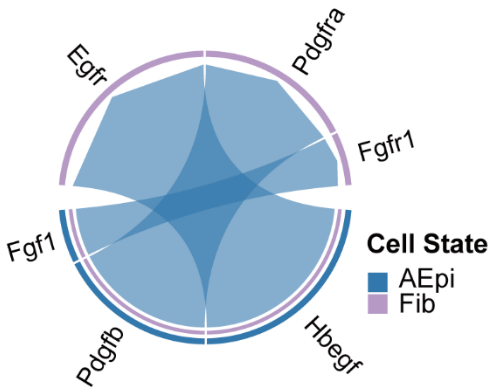

Figure S8 Contributions of LR pairs to EGF, FGF, PDGF, and VEGF signaling pathways from AEpi to fibroblast shown in Figure S7B.

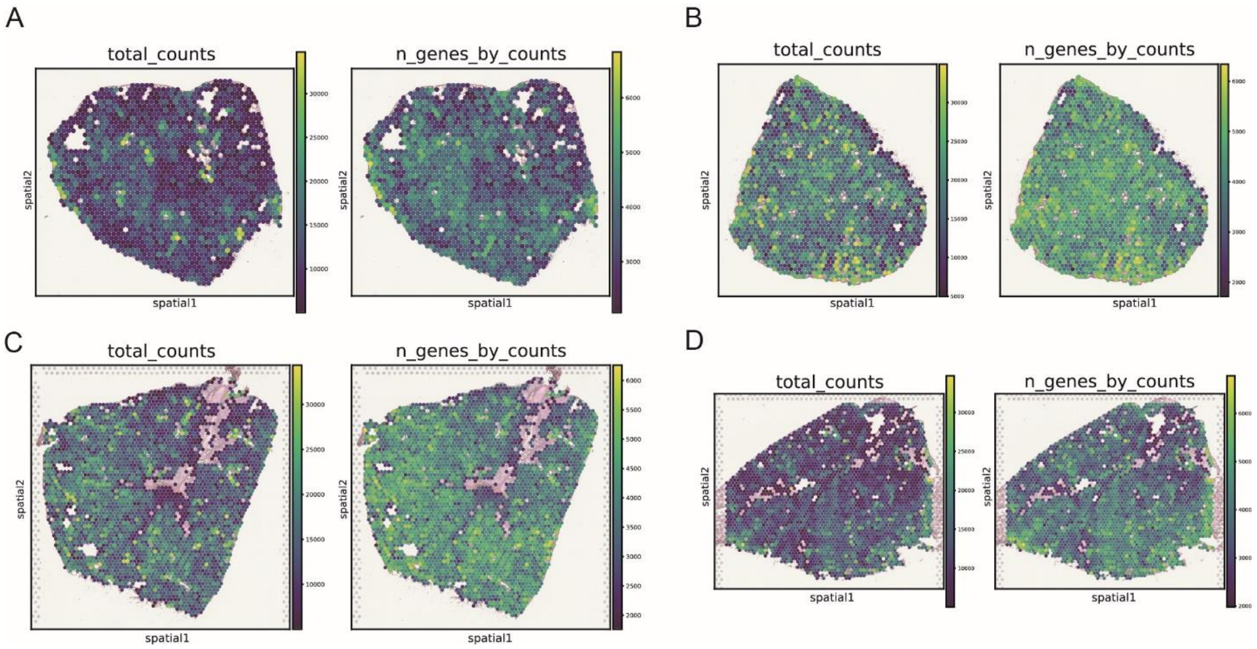

Figure S9 Visualization of total detected counts and genes in spots of the lung tissue sections for 0h (A), 8h(B), 24h(C) and 48h(D) experimental conditions.

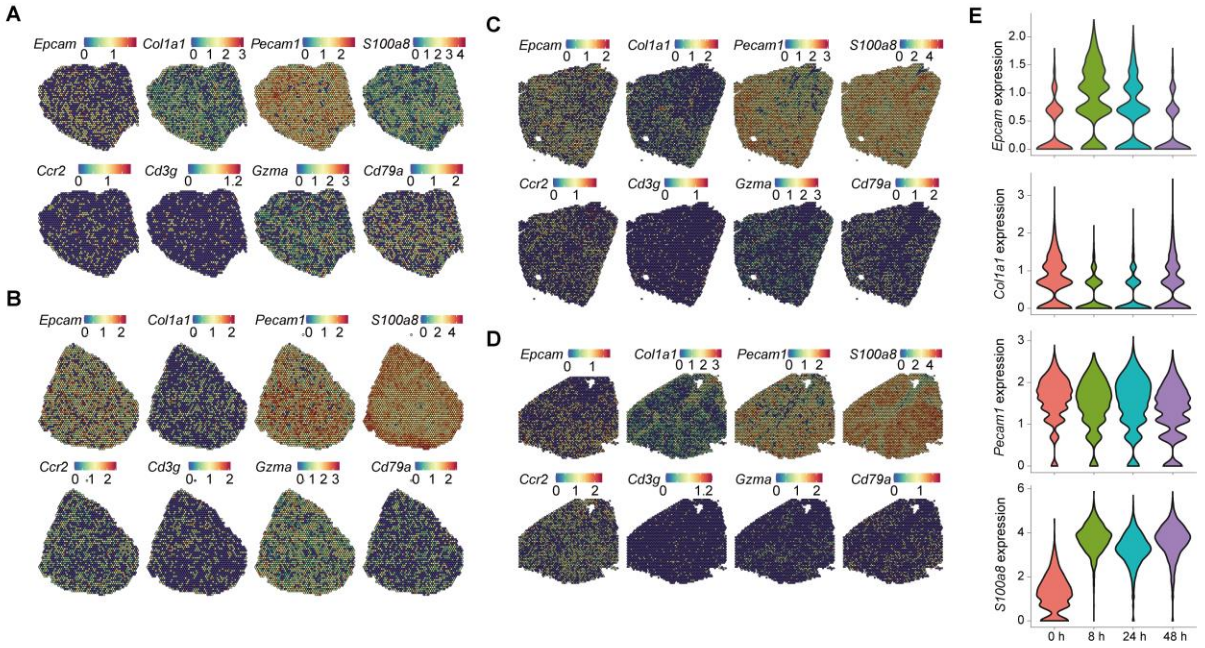

Figure S10 Cell type-specific gene expression signatures of epithelial cell, fibroblast, endothelial cell, and neutrophil in STomics data. (**A**-**D**) Cell type-specific gene expression signatures identify in Figure 1 at 0 h (**A**), 8 h (**B**), 24 h (C), and 48 h (D) tissues after ricin challenge. (**E**) Violin plots showing gene expression profile for the above four cell markers.

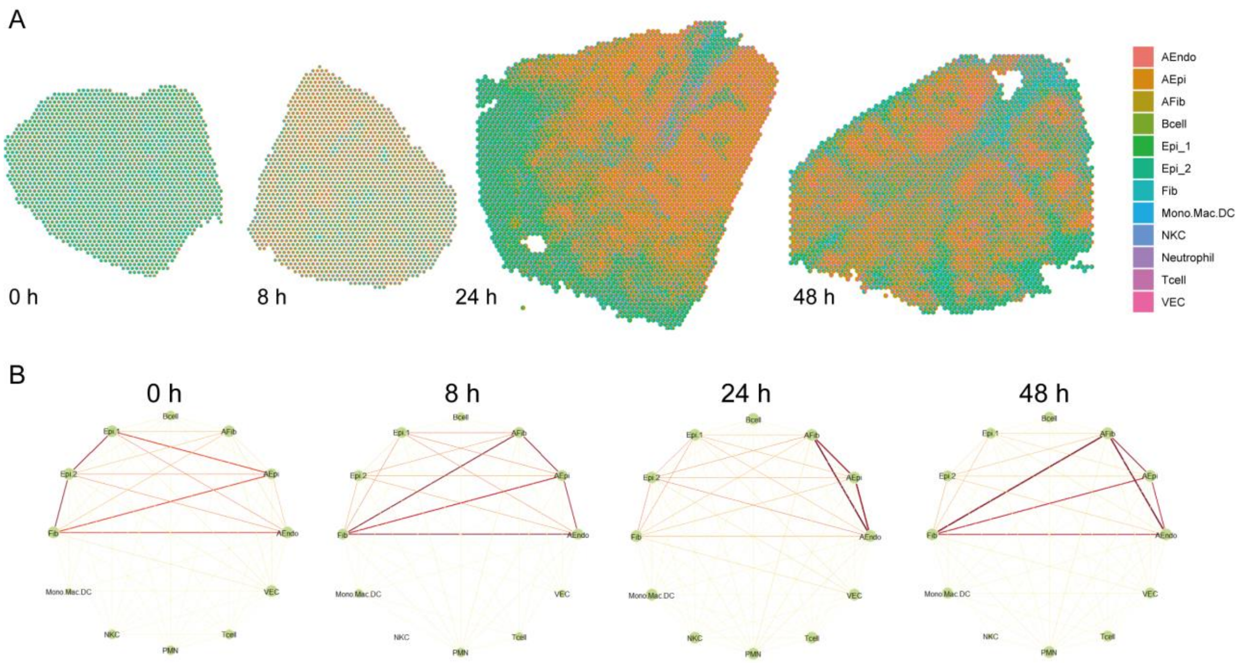

Figure S11 Deconvolution analysis showing the spatial distribution of distinct cell type from scRNA-seq (A) and the correlation profile between cell types (B).

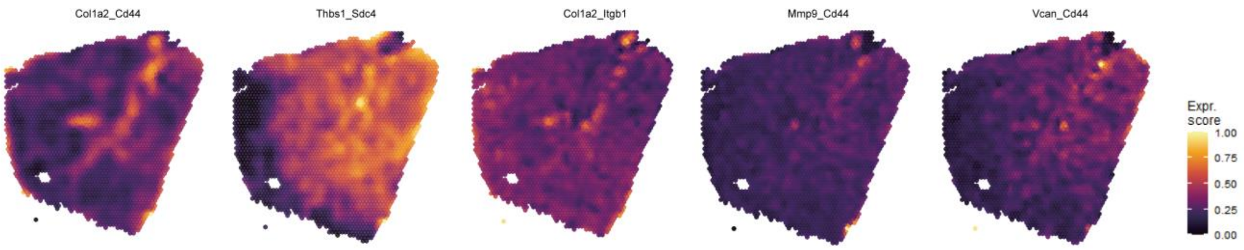

Figure S12 Spatially resolved gene expression for cell-cell interaction associated genes.
